# Dosimetric assessment of patient dose calculation on a deep learning‐based synthesized computed tomography image for adaptive radiotherapy

**DOI:** 10.1002/acm2.13595

**Published:** 2022-03-25

**Authors:** Olga M. Dona Lemus, Yi‐Fang Wang, Fiona Li, Sachin Jambawalikar, David P. Horowitz, Yuanguang Xu, Cheng‐Shie Wuu

**Affiliations:** ^1^ Department of Radiation Oncology Columbia University Irving Medical Center New York City New York USA; ^2^ Department of Radiology Columbia University Irving Medical Center New York City New York USA; ^3^ Herbert Irving Comprehensive Cancer Center New York City New York USA

**Keywords:** deep learning, dosimetric accuracy, synthesized CT

## Abstract

**Purpose:**

Dose computation using cone beam computed tomography (CBCT) images is inaccurate for the purpose of adaptive treatment planning. The main goal of this study is to assess the dosimetric accuracy of synthetic computed tomography (CT)‐based calculation for adaptive planning in the upper abdominal region. We hypothesized that deep learning‐based synthetically generated CT images will produce comparable results to a deformed CT (CTdef) in terms of dose calculation, while displaying a more accurate representation of the daily anatomy and therefore superior dosimetric accuracy.

**Methods:**

We have implemented a cycle‐consistent generative adversarial networks (CycleGANs) architecture to synthesize CT images from the daily acquired CBCT image with minimal error. CBCT and CT images from 17 liver stereotactic body radiation therapy (SBRT) patients were used to train, test, and validate the algorithm.

**Results:**

The synthetically generated images showed increased signal‐to‐noise ratio, contrast resolution, and reduced root mean square error, mean absolute error, noise, and artifact severity. Superior edge matching, sharpness, and preservation of anatomical structures from the CBCT images were observed for the synthetic images when compared to the CTdef registration method. Three verification plans (CBCT, CTdef, and synthetic) were created from the original treatment plan and dose volume histogram (DVH) statistics were calculated. The synthetic‐based calculation shows comparatively similar results to the CTdef‐based calculation with a maximum mean deviation of 1.5%.

**Conclusions:**

Our findings show that CycleGANs can produce reliable synthetic images for the adaptive delivery framework. Dose calculations can be performed on synthetic images with minimal error. Additionally, enhanced image quality should translate into better daily alignment, increasing treatment delivery accuracy.

## 1 INTRODUCTION

Implementation of adaptive radiation therapy demands complex and fast treatment delivery verification techniques that consider different uncertainties in dose delivery. Among the greatest contributors to uncertainties during treatment are the inter‐fractional anatomical changes due to organ motion and tumor size changes. A systematic feedback of images acquired during daily treatment is required to monitor treatment variation and to re‐optimize or adapt the treatment plan during the course of treatment.

Due to high inter‐fraction anatomical variability, liver and pancreatic cancer patients are ideal candidates to benefit from adaptive radiation therapy. Daily cone beam computed tomography (CBCT) is commonly acquired for these patients to rigidly align the target volume to the planning computed tomography (CT). However, due to internal organ motion a deformable image registration‐based alignment is more suitable to accurately adapt the treatment plan to the new geometry. Furthermore, to precisely adapt the treatment plan to these daily variations, it is necessary to recalculate the daily dose to be delivered.^1^

Dose computation using CBCT images is inaccurate for the purpose of treatment planning. The cone‐shaped beam used in CBCT images significantly increases photon and electron scattering, reducing the accuracy of the conversion from Hounsfield units (HU) to relative electron density. Previous estimates comparing the dose calculated from CBCT with planning CTs report up to 3% discrepancy.^2^ Thus, correction of the CBCT images is required before attempting adaptive radiation therapy implementation.

In the past 30 years, different strategies have been implemented to correct relative electron density values from CBCT HU values. The first attempts to correct CBCT images were based on analytical methods where the CBCT signal was defined as a convolution of the primary signal and a scatter kernel. The main issue with this technique was on the estimation of the true scatter kernel.^3^, ^4^ Analytical methods have yielded improved image quality achieving a 2% error^4^; however, scatter estimation accuracy is still insufficient under high heterogeneous conditions. Monte Carlo‐based methods have been more robust in simulating scatter distribution, yet routine application has been limited due to the low computational efficiency among other issues.^5^ Histogram matching is yet another method to correct CBCT images. This method works best for patient‐specific histograms with a 0.9% error^6^ or slice‐specific histograms^7^ with a 3% error. Histogram matching methods rely on having paired datasets. When CBCT‐based anatomy diverges from CT‐based anatomy, the accuracy of the method turns out to be significantly reduced.

Currently, the most common method to perform CBCT‐based patient dose calculation consists of deforming the planning CT to target daily CBCT anatomy. Dose calculation is performed in the deformed CT (CTdef) preserving the accuracy of the HU to electron density. This method is based on image registration, which consists of aligning homologous points in a source and a target image. Any image registration algorithm contains a transformation model describing the allowed degrees of freedom, a similarity metric that quantifies the source to target alignment, and an optimization routine to find the transformation that maximizes similarity. The main limitation of this method usually arises from the transformation model that also defines the maximum allowed deformation. Large deformation of internal organs generally produces incorrect outer contours that affect the accuracy of the dose calculation.^1^, ^8^

With the current advances in artificial intelligence and deep learning techniques, novel methods to correct for CBCT images have been proposed. Translational research from image processing and image reconstruction fields have created unsupervised image to image translation systems capable of learning the optimal transformation between two image sets. Furthermore, translation systems where the source and target images are un‐paired have also been implemented.^9^ The latter is relevant in adaptive radiation therapy because daily CBCT images usually do not have a paired CT image. Generative adversarial networks (GANs) have been successfully used in the un‐paired image‐to‐image translation. This unsupervised machine learning method consists of two neural networks, a generator and a discriminator. The generator's goal is to produce synthesized images that can fool the discriminator while the discriminator's goal is to distinguish synthesized images from real images. This process repeats until the generator is fully trained in creating synthetic images that the discriminator is unable to differentiate from the real ones. The accuracy of the synthesized image is ultimately controlled by a set of loss functions which are continuously being minimized.

A previous study by Zhao et al.^10^ have used GANs to correct ring artifacts in CBCT images. This study combined the smooth loss with the generative adversarial loss producing a generator‐discriminator pair capable of creating synthesized CBCT images without ring artifacts. Furthermore, a paper by Liang et al.^11^ implemented a cycle‐consistent GAN methodology to generate synthesized CT images from CBCT images for head and neck cases. That study also compared the synthesized CT images with deformed planning CT images showing comparative results in terms of HU accuracy and dose distribution.

Our main objective is to assess the dosimetric accuracy of a synthetic CT‐based calculation for adaptive planning. In terms of dose distribution, we hypothesize that synthetically generated images will produce comparable results to a CTdef in terms of dose calculation, while displaying a more accurate representation of the daily anatomy and therefore superior dosimetric accuracy. To achieve this goal, we implemented a GAN to generate synthetic CT images in an anatomy with higher inter‐fraction variability such as the upper abdominal cavity. A verification plan was created to calculate the dose distribution using the synthesized image and the results were compared with a CTdef‐based calculation and a CBCT‐based calculation corresponding to the first treatment fraction.

## 2 MATERIALS AND METHODS

### 2.1 Image acquisition and preprocessing

The process for creating the synthetic CT images, containing anatomical information from the CBCT images and the correct HU from the planning CT, requires two training cycles: CBCT‐to‐CT and CT‐to‐CBCT.

The planning CT images and CBCT images corresponding to the first treatment fraction were retrospectively obtained from 17 patients who underwent stereotactic body radiation therapy (SBRT) for hepatocellular carcinoma. These were used in the first training step to create the planning CT datasets and the CBCT datasets, respectively. All patient images were stripped of all personal identifiers for anonymization and stored internally according to our institution protocol (IRB 888S5260). Data from 11 patients (1760 images) were used for training the deep learning algorithm while the images from six patients were used for testing (616 images). Data augmentation techniques were used to amplify fourfold the training set to a total of 7040 images. Classic 10‐fold cross‐validation was used to evaluate and fine‐tune our model. We chose a relatively low amount of validation data due to limited data availability and to maximize the data available for training.

The planning CT images were acquired on a Siemens Definition AS20 with a resolution of 1.2695 × 1.2695 × 2 mm (512 × 512 × 246 pixels), 120 kVp, 142 mA, while the CBCT images were acquired on a Varian TrueBeam On‐Board Imager with a resolution of 0.908 × 0.908 × 1.988 mm (512 × 512 × 88 pixels), 125 kVp, 20 mA. All CT images were rigidly registered, resampled, and resized to match the size and resolution of the CBCT images before starting the training stage.

Deformable registered CTs (CT‐to‐CBCT, CTdef) was created for the testing dataset to assess HU and dose calculation accuracy of the synthetic CT. The CTdef was created in Velocity (Varian Medical Systems, Inc., Palo Alto, CA) using the multistep registration tool, as this tool is commonly used in the clinical setting.

The environment used to implement the deep learning algorithm consisted of four NVIDIA 2080Ti graphics processing units (GPUs) each with 11 GB of GPU RAM with an Intel CPU Core i7 7820X, 128 GB of system memory running Ubuntu, Anaconda, Python 3.6.6 and Chainer 5.0.0. The training phase consisting of 50 epochs required approximately 12 h and the testing phase achieved a translation speed of 64 images per second.

## 3 CYCLE‐CONSISTENT GENERATIVE ADVERSARIAL NETWORK OVERVIEW

**FIGURE 1:**
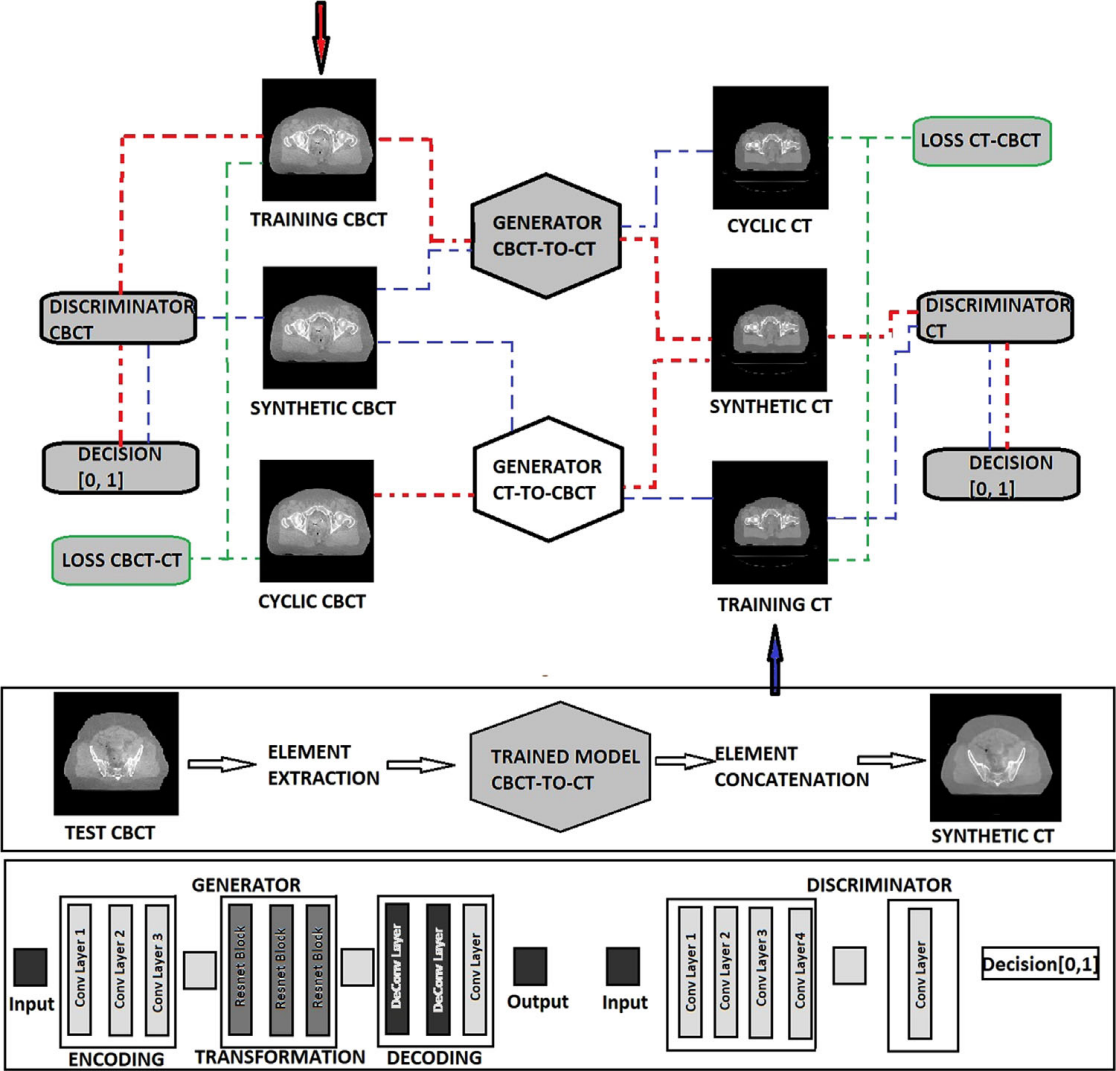
Cycle‐consistent generative adversarial network (CycleGAN) training and testing architecture. The red arrow initiates training for cone beam computed tomography (CBCT) to computed tomography (CT) image translation while the blue arrow initiates training for CT‐to‐CBCT image translation. The generator and discriminator structures are shown in the bottom panel.

For this study, we used the Chainer^12^ implementation of cycle‐consistent generative adversarial network (CycleGAN) proposed by Zhu et al.^9^ combined with the loss function optimization proposed by Kida et al.^13^ Figure 1 shows the schematics of the CycleGAN architecture we used in this study. In the training stage, the goal is to train two generators: CBCT‐to‐CT (G_CBCT→CT_) and CT‐to‐CBCT (G_CT→CBCT_) and two discriminators: CBCT discriminator (D_CBCT_) and CT discriminator (D_CT_). The two cycles in the training architecture are represented by the red and blue lines, respectively. The cyclic images are created by running a training image through a generator and its inverse (G_CBCT→CT,_ G_CT→CBCT_). The difference between the original image and the cyclic image must approach zero as the training cycles progress. At this point the loss function is calculated.

The generators have multiple layers that perform three computational phases: (1) encoding the input image using convolutional neural network (cNN) layers that extract the image features; (2) transforming the features by passing them through three layers of residual blocks; and (3) decoding the transformed features using transposed cNN layers to build an output image with the same size as the input image.

The loss functions define the goals we want to achieve with the mapping. In this case, our goal was to preserve the anatomical characteristics of the CBCT images and the pixel intensity or HU number of the CT images. Table 1 shows the loss functions and hyper‐parameters that were implemented in our algorithm to optimize the synthesis of a CT image from a CBCT image. The stochastic gradient‐descent method was used to minimize the objective functions. Additional details on the loss functions can be found in the Supporting Information.

**TABLE 1:** Summary of loss functions and hyper‐parameters

Functions	Purpose	Hyper‐parameters	Value
loss_cycle_	Cycle consistency loss, G_CBCT→CT _= G_CT→CBCT_^’^	λ_cycle_	10.0
loss_adv_	Adversarial loss, synthetizes realistic images	λ_adv_	1.0
loss_grad_	Gradient loss, preserves edges and structures	λ_grad_	1.0
loss_idem_	Idempotent loss, idempotency preservation	λ_idem_	1.0
loss_tv_	Total variation regularization loss, produce spatially uniform images	λ_tv_	1.0

### 3.1 Implementation modifications

GAN algorithms were not originally designed for medical images, therefore they usually run out of GPU memory when dealing with 512 × 512 images. To overcome this issue, we cropped the images to a 480 × 384 size, removing most of the free space (air). At 16 bits of depth, the maximum size we were able to load on memory was 256 × 256, therefore, the space was randomly sampled four times with a 256 × 256 matrix. This approach augmented the original data, improving the accuracy of the training process.

Despite previous studies^13^ recommending the use of a reduced HU range to train the algorithm, a full HU range was used for this implementation. Narrowing the HU range to fit tissue variability generally improves the optimization. The CBCT and CT images for liver and pancreas include significant portions of the lungs. Therefore, a larger range of HU from −1000 HU to 3000 HU was considered in the training phase.

### 3.2 Assessment metrics

Accuracy of the synthetically generated images was assessed considering the preservation of anatomical structures, image quality, and HU accuracy. We used well established metrics such as the signal‐to‐noise ratio (SNR), the root mean square error (RMSE), and the mean absolute error (MAE) to assess the image quality of the synthetic image (*t*) compared to the reference image (*r*) (Equations (1), (2), and 3). The checkerboard method, Hausdorff distance, and Dice similarity metrics were used to assess fine image structures and edge matching between registered images and pixel intensity histograms (Equations 4 and 5). Organ‐specific statistics were used to assess HU accuracy.

The dosimetric accuracy of the synthetic‐based dose calculation was assessed using dose volume histograms (DVHs), isodose lines comparison, and the gamma index proposed by Low et al.^14^

(1) $$\mathrm{SNR}=10\cdot log_{10}\left[\frac{\sum_{0}^{n_{x}-1}\sum_{0}^{n_{y}-1}{\left[r\left(x,y\right)\right]}^{2}}{\sum_{0}^{n_{x}-1}\sum_{0}^{n_{y}-1}{\left[r\left(x,y\right)-t\left(x,y\right)\right]}^{2}}\right]$$

(2) $$\mathrm{RMSE}=\sqrt{\frac{1}{n_{x}n_{y}}\sum_{0}^{n_{x}-1}\sum_{0}^{n_{y}-1}{\left[r\left(x,y\right)-t\left(x,y\right)\right]}^{2}}\;$$

(3) $$\mathrm{MAE}=\frac{1}{n_{x}n_{y}}\sum_{0}^{n_{x}-1}\sum_{0}^{n_{y}-1}{\left[r\left(x,y\right)-t\left(x,y\right)\right]}^{2}$$

(4) $$\begin{array}{ccc} & & Hausdorff\;distance\;\left(X,Y\right)\\ & & =\;\max\left(\mathrm{ma}x_{x\in X}\left(\mathrm{mi}n_{y\in Y}\left\|x-y\right\|\right),\right.\\ & & \left.\mathrm{ma}x_{y\in Y}\left(\mathrm{mi}n_{x\in X}\left\|y-x\right\|\right)\right) \end{array}$$

(5) $$\mathrm{Dice}\left(X,Y\right)=\frac{2\left|X\cap Y\right|}{\left|X\right|+\left|Y\right|}$$

**FIGURE 2:**
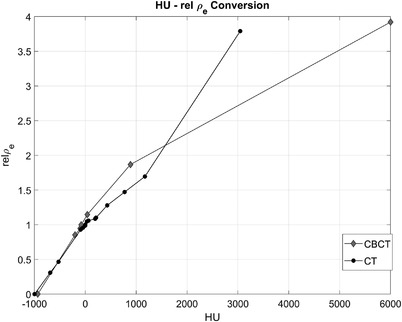
Planning computed tomography (CT) and cone beam computed tomography (CBCT) Hounsfield unit (HU)—relative electron density calibration curves. The CT calibration is the standard curve used for clinical purposes. The CBCT was measured for comparison purposes.

The synthetic‐based dose calculation was done with the same calibration curve (HU – electron density) as the planning CT. This calibration corresponds to the CT Siemens simulator. A calibration curve was created for the CBCT images acquired in the Varian On‐Board Imager (OBI). The OBI calibration curve was created using the Catphan ®504 phantom with the half‐fan filter to measure HU values for different materials (Table 2). The OBI calibration curve as shown in Figure 2 was added to the beam configurations before dose calculation.

**TABLE 2:** Hounsfield unit (HU) measurements of the Catphan ®504 phantom materials

Material	HU‐measured	Relative electron density^a^	Mass density (g/cm^3^)
Acrylic [C5H8O2]	40	1.147	1.180
Air [.78N,.21O,.01Ar]	−934	0.001	0.001
Polystyrene^b^ [C8H8]	−81	0.998	1.050
LDPE^c^ [C2H4]	−100	0.945	0.904
PMP [C6H12(CH2)]	−202	0.853	0.830
Teflon [CF2]	888	1.868	2.200
Max	6000/13 520^d^	3.920	9.500

^a^ Relative electron density is the electron density of the material in e/cm^3^ divided by the electron density of water (H_2_O) in e/cm^3^.

^b^ Polymethylpentene.

^c^ Low density polyethylene.

^d^ Values taken from the Eclipse standard curve. Relative electron density/mass density.

## 4 RESULTS

### 4.1 Accuracy of the synthetic image

Preservation of anatomical structures, image quality, and HU's accuracy were the three measures used to assess the accuracy of the synthetic image. The synthetically generated image was compared with the planning CT image and the CBCT image in terms of these measures. Preprocessing steps were necessary such as a rigid registration transformation to center both images at the same anatomical level and to crop the excess CT slices from 480 to 88 slices.

#### 4.1.1 Image quality

Basic image quality of the synthetic image was assessed by computing SNR, RMSE, and MAE. The planning CT image was used as the reference image for calculation purposes. Table 3 shows that the synthetic images have lower SNR and lower RMSE and MAE when compared with the CBCT image.

**TABLE 3:** SNR, RMSE, and MAE for the synthetic image and the CBCT image. The CT image was used as the reference image

Image		SNR (dB)	RMSE (HU)	MAE (HU)
CBCT	P‐1	8.79	107.80	81.31
	P‐2	0.97	112.02	74.31
	P‐3	2.04	138.14	71.10
	P‐4	3.44	156.81	66.00
	P‐5	0.58	164.77	80.88
	P‐6	2.05	144.24	64.15
	Mean	2.98 ± 2.75	137.29 ± 21.19	72.95 ± 6.63
Synthetic	P‐1	6.16	25.64	19.86
	P‐2	0.09	99.18	51.56
	P‐3	1.76	123.88	60.11
	P‐4	1.84	130.39	62.39
	P‐5	0.28	154.06	70.23
	P‐6	0.41	119.44	62.47
	Mean	1.76 ± 2.09	108.765 ± 40.54	54.44 ± 16.39

Abbreviations: CBCT, cone beam computed tomography; CT, computed tomography; HU, Hounsfield unit; MAE, mean absolute error; RMSE, root mean square error; SNR, signal‐to‐noise ratio.

**FIGURE 3:**
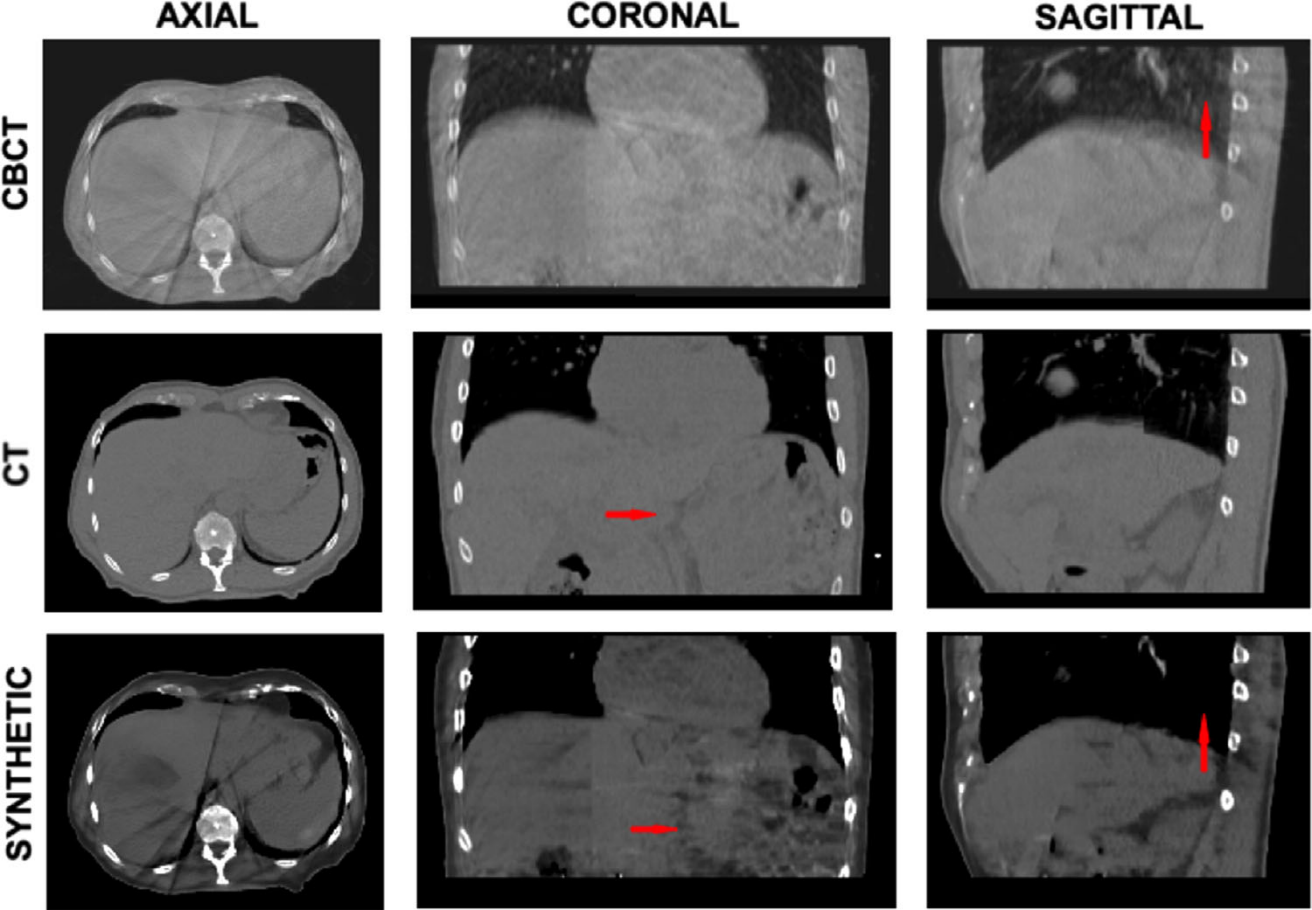
Image quality visualization. Cone beam computed tomography (CBCT) (top W:541 L:116), computed tomography (CT) (middle W:541 L:116), and synthetic CT (bottom W:541 L:116) images from the patient of interest (test patient). Axial, coronal, and sagittal slices from left to right in that order. Red arrows on the sagittal and coronal slices pointing at lung artifact reduction and improved resolution, respectively.

As shown in Figure 3, synthetically generated images have higher contrast resolution and lower noise and artifacts severity than the CBCT. Noise is significantly reduced in the lungs, while an overall increased contrast resolution has been observed. Streak artifacts are still present in the images, albeit significantly reduced. The deep learning algorithm produces images with sharper edges, therefore the HU values in air near air‐tissue interfaces appear lower than the ones expected in a real CT image.

#### 4.1.2 Preservation of anatomical structures

**FIGURE 4:**
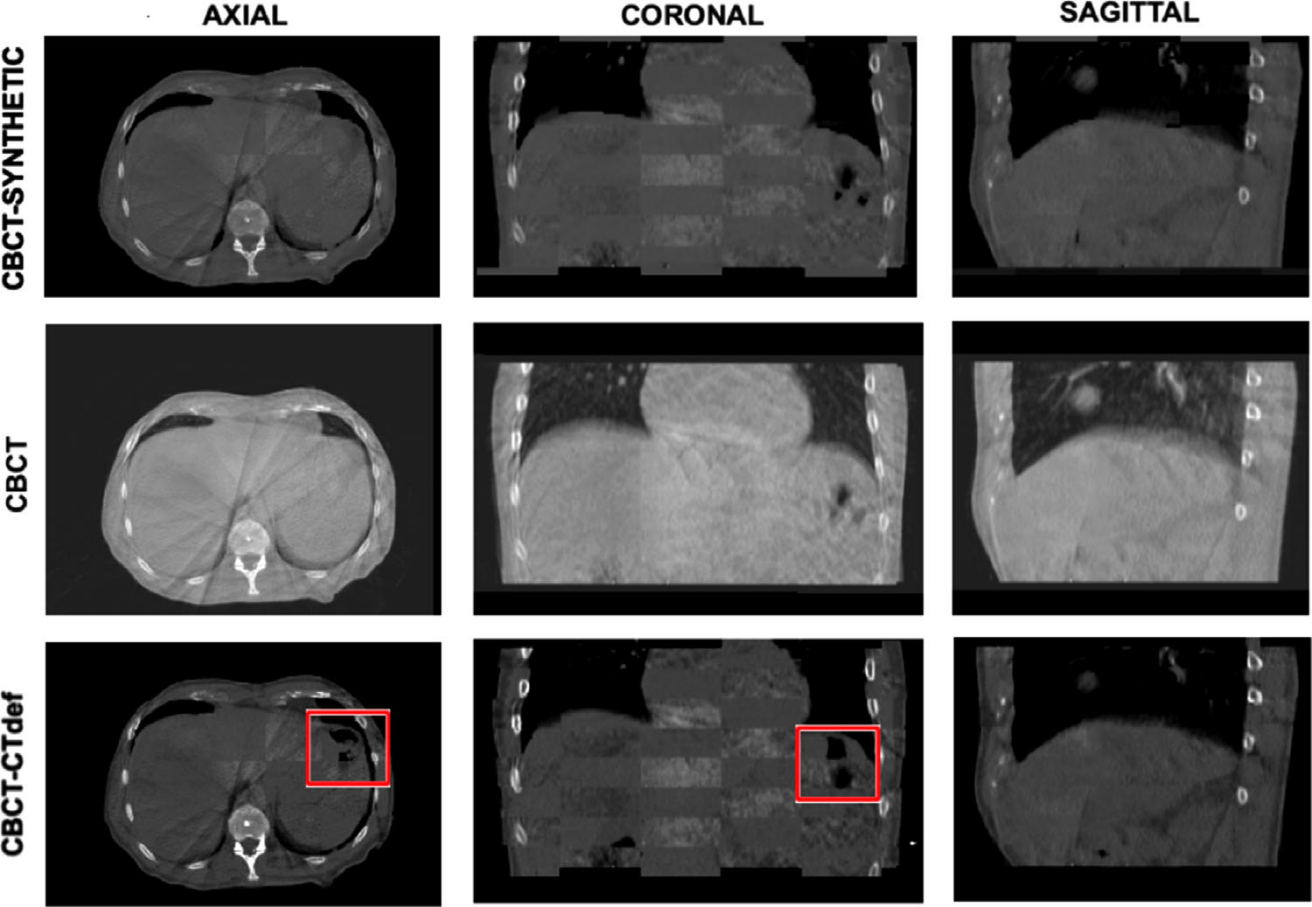
Anatomical preservation. Checkerboard pattern for the synthetic and cone beam computed tomography (CBCT) (top) image, CBCT image (middle), and checkerboard pattern for deformed CT (CTdef) and CBCT (bottom) images. Axial, coronal, and sagittal slices from left to right in that order. Window level and window width of the CBCT image was changed to W:1087 L: −88 with the purpose of better displaying the differences between the overlapped images.

The checkerboard method was used to establish a detailed comparison of fine‐image structures and edge matching between the CBCT and the synthetic image. The images were cut in an 8‐by‐8 checkerboard pattern of squares with the synthetic image and the CBCT image displaying in alternate squares. Additionally, a checkerboard pattern was created for the deformed planning CT (CTdef) to assess the advantage of using a synthetic image as opposed to using the CTdef. A visual comparison between both images is shown in Figure 4. Edge matching and sharpness is well preserved in the synthetic image. The learning algorithm was implemented exclusively with axial slices; however, edge matching is also preserved in the sagittal and coronal planes.

Hausdorff distance metrics and Dice similarity metrics were used to establish a quantitative comparison between structures contoured independently on the synthetic images and the CTdef images using the daily CBCT contours as the reference (Table 4). The contours in the synthetic images are consistently closer to the CBCT contours than the deformed CT contours as shown by the mean Hausdorff distance. Additionally, the Dice coefficient shows a greater correlation between the synthetic contours and the CBCT contours.

**TABLE 4:** Hausdorff distance and Dice similarity metrics (mean ± standard deviation [STD]). The cone beam computed tomography (CBCT) image was used as the reference image

Image		Mean Hausdorff distance (mm)	Dice Coefficient (Coeff)	∆V(cc)
Synthetic	Liver	1.49 ± 0.71	0.97 ± 0.015	6 ± 3
	Heart	1.29 ± 0.60	0.95 ± 0.027	3 ± 2
	Kidney	1.36 ± 0.62	0.91 ± 0.033	8 ± 5
	Spinal canal	0.79 ± 0.46	0.88 ± 0.031	2 ± 1
	Lungs	0.89 ± 0.51	0.87 ± 0.054	5 ± 3
	Mean	1.16 ± 0.27	0.92 ± 0.04	5 ± 2
CTdef	Liver	4.18 ± 2.02	0.87 ± 0.042	63 ± 33
	Heart	2.59 ± 1.21	0.90 ± 0.051	6 ± 4
	Kidney	2.71 ± 1.16	0.82 ± 0.063	15 ± 7
	Spinal canal	1.66 ± 0.08	0.73 ± 0.050	8 ± 7
	Lungs	3.56 ± 1.54	0.79 ± 0.091	52 ± 32
	Mean	3.26 ± 0.65	0.82 ± 0.06	29 ± 24

Abbreviation: CTdef, deformed computed tomography.

While the synthetic images can replicate the anatomy of the CBCT images, the CTdef fails at matching the CBCT image in specifically localized regions. Large spatial variations of gastrointestinal air cavities caused the registration algorithm to fail in achieving an accurate deformable registration around them. Additional details of the process for creating and assessing the deformable registered images are reported in the Supporting Information.

#### 4.1.3 Accuracy of Hounsfield units

**FIGURE 5:**
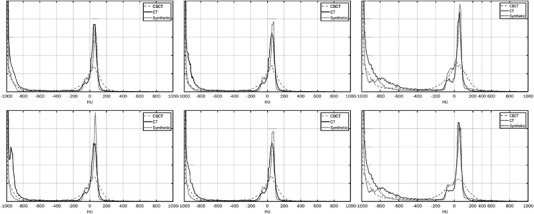
Hounsfield unit (HU) histograms for the testing volumes corresponding to the computed tomography (CT), cone beam computed tomography (CBCT), and synthetic images, respectively. The vertical axis represents the count (frequency) and the horizontal axis represents the HU range.

HU accuracy was assessed globally by plotting histograms of the three‐dimensional (3D) volumes for CBCT, CT, and synthetic images, respectively. As shown in Figure 5, the histogram of the synthetic volumes closely tracks the histogram of the CT volume. The synthetic image histogram shows a better differentiation of soft tissue when compared to the CBCT volume. Perfect match is not reasonably expected as there are anatomical differences across the unpaired datasets. The presence of fiducials on the planning CT as well as differences between the couches also contribute to differences between the synthetic histogram and the CT image histogram.

To address these differences, HU accuracy was assessed for specific organs of interest. The CBCT, CT, and synthetic images were independently contoured to show the aorta, spinal canal, heart, bones, kidneys, lungs, and the liver. Mean and standard deviation values are shown in Table 5. The mean percent HU difference of the synthetic HU values per organ relative to the CT image was 17.2% (max 19%—kidneys; min 4.5%—spinal canal). Under the same conditions, the mean percent HU difference of the CBCT HU values per organ, relative to the CT image was 42.8% (max 105%—heart; min 10.3%—bones). Additionally, overall variability of the synthetic image correlates with the CT image variability. CBCT HU variability is higher than the CT variability for soft tissue, while the overall variability is lower than CT for bone and air.

**TABLE 5:** Hounsfield units (HU) mean, standard deviation (STD), and segment HU variability $\left(\sqrt{\sum_{n\;=\;1}^{n\;=\;6}{\sigma_{\mathrm{Pn}}}^{2}}\right)$ for specific regions of interest across test subjects

	CT			CBCT			Synthetic		
Segments	Mean	STD	$\sqrt{\sum_{n\;=\;1}^{n\;=\;6}{\sigma_{Pn}}^{2}}$	Mean	STD	$\sqrt{\sum_{n\;=\;1}^{n\;=\;6}{\sigma_{Pn}}^{2}}$	Mean	STD	$\sqrt{\sum_{n\;=\;1}^{n\;=\;6}{\sigma_{Pn}}^{2}}$
Aorta	40	4	29	63	14	101	50	1	29
Spinal canal	22	6	34	26	2	79	23	9	43
Heart	36	2	32	74	13	75	42	7	33
Bones	349	64	250	385	44	200	400	21	246
Kidney	21	7	33	8	12	159	25	5	26
Lungs	−719	11	225	−600	97	222	−804	24	191
Liver	54	3	28	38	13	70	57	1	48

Abbreviations: CBCT, cone beam computed tomography; CT, computed tomography.

### 4.2 Dosimetric accuracy of synthetic‐based treatment planning

The planning CT image was deformed to the CBCT of the first treatment fraction to create a reference image with the purpose of establishing the accuracy of the synthetic‐based dose calculation. Details and assessment of the deformable registration process are described in the Supporting Information. Overall, the body contour and the bones match the CBCT image; however, structures such as the liver, heart, and stomach show target registration errors up to 7 mm. Aliasing artifacts were also present on the CTdef images.

The synthetic images and the CTdef of the test patients were loaded into Eclipse (Varian Medical Systems) treatment planning system. Three verification plans were created maintaining the same monitor units according to the original treatment plan. Acuros XB version 13.6.23 (Varian Medical Systems) was used as the calculation model with 1 mm grid size. The test patients had 5000 cGy in five fractions prescribed for the treatment of a liver metastasis with 95% of the planning target volume (PTV) covered by 95% of the prescription dose. The treatments were optimized for three volumetric‐modulated arc therapy (VMAT) partial arcs, 6MV energy on the Truebeam.

#### 4.2.1 Dose volume histograms analysis

DVHs are the most common plan evaluation tools to compare doses from different plans to tissue volumes. All PTVs and organs at‐risk were calculated for the three plans: CTdef, CBCT, and synthetic. To ease the visualization process, we exclusively included in the plots the PTV and the liver. All three histograms were calculated on the CBCT contours for each testing subject.

**FIGURE 6:**
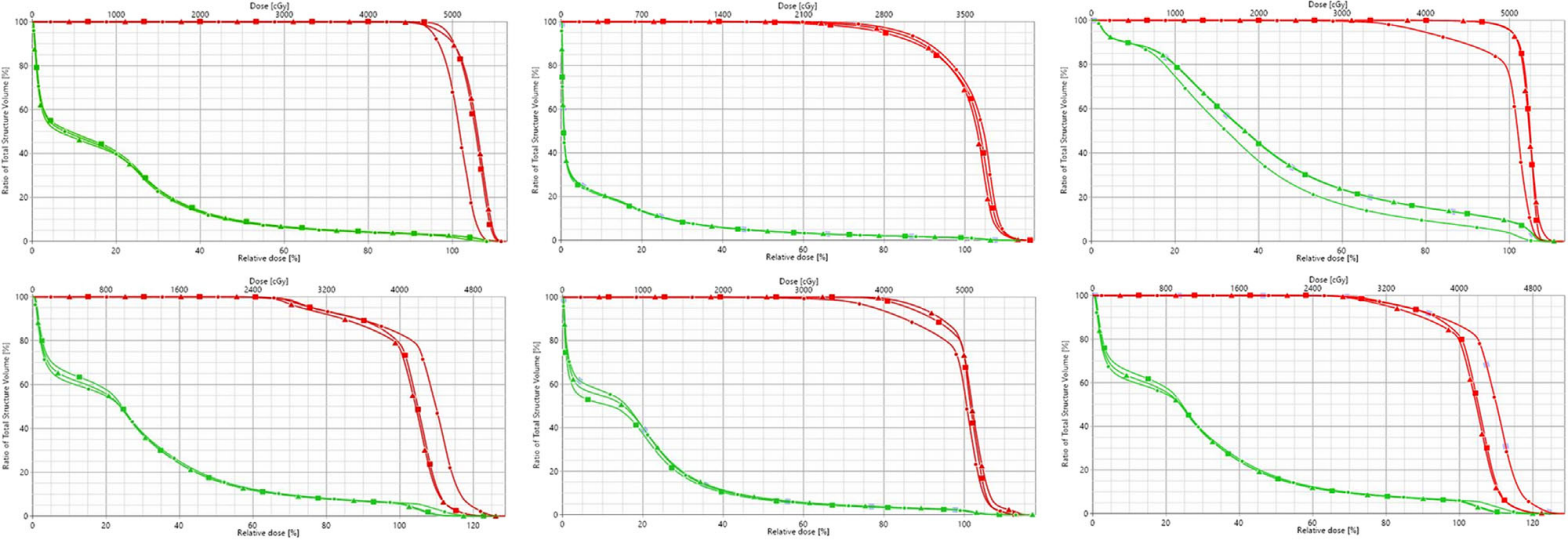
Cumulative dose volume histograms (DVHs) for cone beam computed tomography (CBCT) (circle), synthetic (triangle), and deformed computed tomography (CTdef)‐based (square) dose calculations. Includes the planning target volume (PTV) (red) and the liver (green) as the closest most common organ at‐risk.

The greatest difference between the three plans was observed in the PTV structure. On average, the volume covered at least by 100% of the prescription dose was 92% for CTdef, 93% for synthetic, and 70% for CBCT (Figure 6). Additional dose statistics per volume are reported in Table 6 for the CBCT, CTdef, and synthetic‐based calculation, respectively. The synthetic‐based calculation shows comparatively similar results to the CTdef‐based calculation with a mean deviation of 1.5% to the PTV, while the CBCT‐based calculation showed a mean deviation of 3.6%.

**TABLE 6:** Dose statistics for CBCT, CTdef, and synthetic image

		CBCT‐dose (%)			Synthetic‐dose (%)			CTdef‐dose (%)		
P	Structures	Min	Mean	Max	Min	Mean	Max	Min	Mean	Max
P‐1	PTV	84.1	101.4	112.0	85.4	105.0	111.6	81.8	105.1	113
	Liver	0.1	18.7	109.3	0.1	19.4	111.6	0.1	18.8	112.3
P‐2	PTV	36.7	100.3	117.2	39.3	100.2	115.3	52.7	101.8	115.1
	Liver	0.0	8.1	114.9	0.1	8.4	114.2	0.1	8.3	113.7
P‐3	PTV	28.4	99.4	112.7	74.8	104.5	113.2	66.1	104.4	109.8
	Liver	1.3	37.6	111.1	1.3	43.5	112.8	1.4	43.3	109.8
P‐4	PTV	51.2	101.3	126.7	56.4	102.3	126.3	53.4	106.7	128.7
	Liver	0.1	27.9	123.2	0.3	28.5	123.5	0.4	28.3	127.2
P‐5	PTV	36.5	98.8	115.2	71.5	101.2	117.8	68.4	100.3	114.3
	Liver	0.0	18.8	115.2	0.1	18.7	117.8	0.2	17.3	114.3
P‐6	PTV	51.5	102.5	126.7	59.1	103.4	126.3	53.4	107.9	128.7
	Liver	0.1	27.9	126.2	0.3	28.5	126.5	0.4	28.3	128.2

Abbreviations: CBCT, cone beam computed tomography; CT, computed tomography; CTdef, deformed computed tomography; P, patient; PTV, planning target volume.

#### 4.2.2 Isodose lines comparison

**FIGURE 7:**
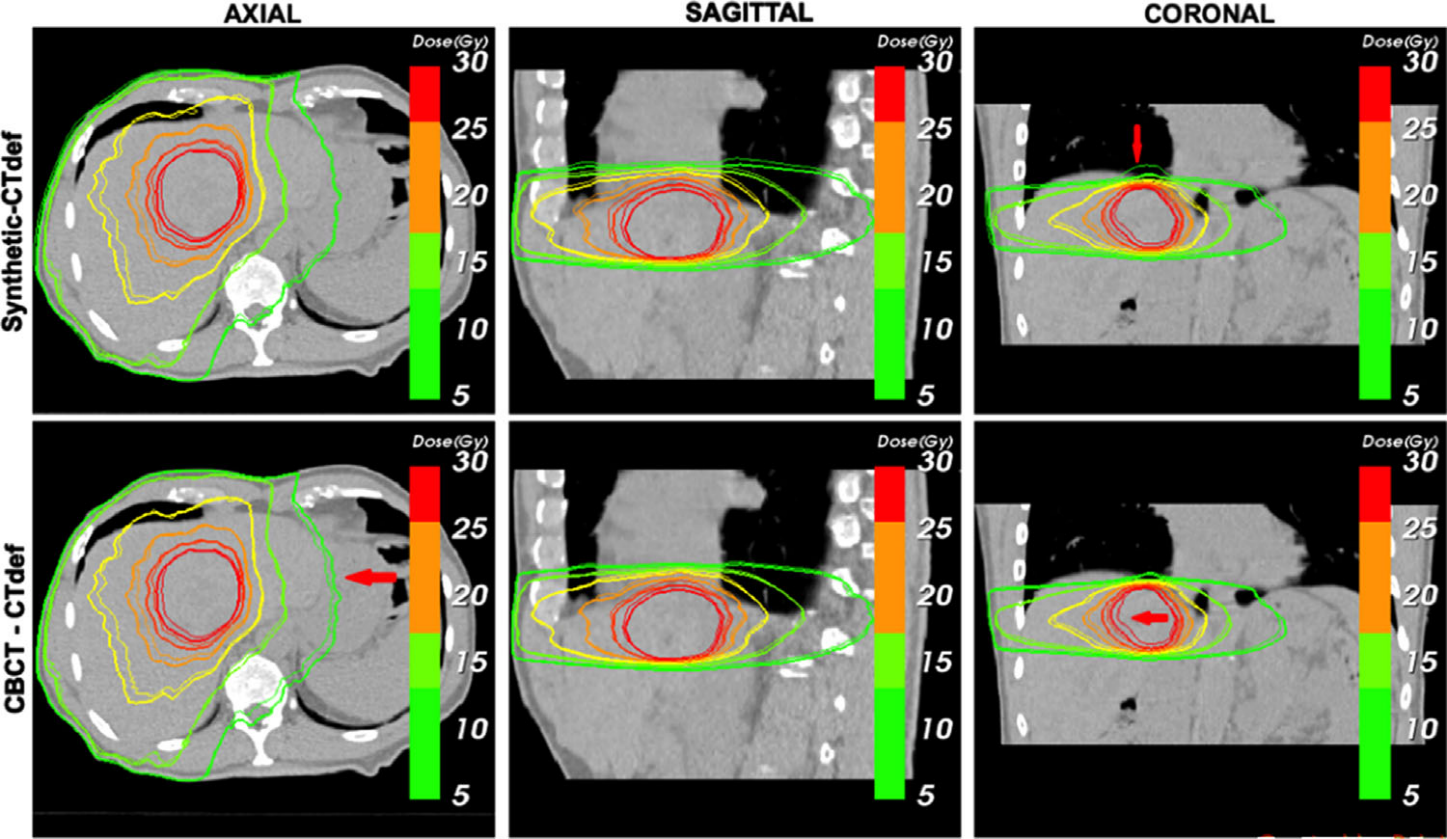
Isodose lines comparison superimposed to the deformed computed tomography (CTdef) image. The thick solid line corresponds to the reference computed tomography (CT)‐based dose calculation while the thin solid line corresponds to the synthetic‐based isodose (upper) and the cone beam computed tomography (CBCT)‐based isodose (lower). Red arrow points to regions of significant divergence (*d* > 2 mm).

**FIGURE 8:**
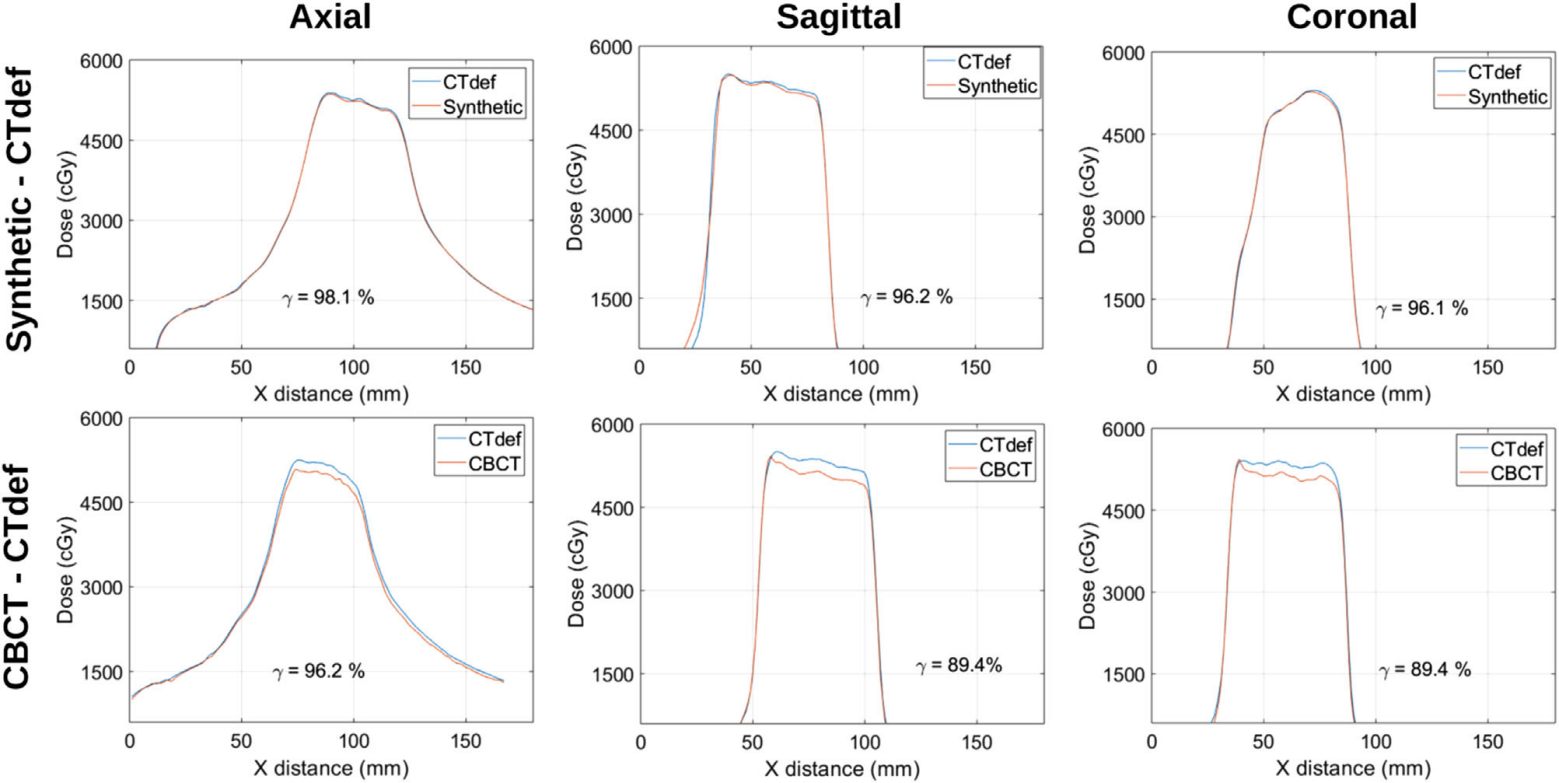
Dose profile comparisons for the lateral direction of the axial, sagittal, and coronal planes respectively for Patient 1.

The main limitation of DVHs is that they offer no spatial information of the dose distributions. To account for spatial differences, we compared axial, sagittal, and coronal isodose distributions for the three calculation methods. Figure 8 shows a comparison between the synthetic‐based calculation and the CTdef isodose lines and a comparison between the CBCT‐based calculation and the CTdef isodose lines of one of the test subjects. Regions with significant deviations (*d* > 2 mm) from the CTdef are marked with a red arrow. Overall, synthetic‐based isodose lines show better agreement to the CTdef isodose lines than the CBCT. In cases where the PTV was near an air‐tissue interface, we observed a disagreement in the low dose region. Figure 7 shows an example of this occurrence near the right lung‐liver interface on the 5 Gy isodose line. This region is commonly blurred due to motion artifacts, with the averaging effect being more pronounced on the CBCT image. The deep learning algorithm produces images with sharper edges, therefore the HU values in air near air‐tissue interfaces are lower than the ones expected in a real CT image.

#### 4.2.3 Two‐dimensional gamma analysis

The gamma comparison is commonly used to quantitatively compare two dose distributions by combining dose difference (∆D) and a distance to agreement (∆d) criteria as proposed by Low et al. (Equation 6). We exported orthogonal slices at the isocenter for the three dose verification plans (CBCT, CTdef, and synthetic) and calculated the two‐dimensional (2D) gamma for a 3%/2 mm, 10% dose threshold criteria using CTdef as the reference dose distribution. Analogous points (*r_e_* and *r_r_*) in the evaluated and reference distributions are said to agree when gamma ≤ 1 where:

(6) $$\begin{array}{ccc} \Gamma \;\left(\vec{r_{e}},\vec{r_{r}}\right) & & =\sqrt{\frac{{\left|\vec{r_{e}}-\vec{r_{r}}\right|}^{2}}{\Delta d^{2}}+\frac{{\left|D\left(\vec{r_{e}}\right)-D\left(\vec{r_{r}}\right)\right|}^{2}}{\Delta D^{2}}},\gamma \;\left(\vec{r_{r}}\right)\\ & & =\;min\left\{\vec{\Gamma }\left(\vec{r_{e}},\vec{r_{r}}\right)\right\}\forall \vec{r_{e}} \end{array}$$

Figure 8 shows dose profile comparisons for the lateral direction of the axial, sagittal, and coronal planes, respectively, for patient 1. The synthetic‐based calculation shows comparatively similar results to the CTdef‐based calculation with a mean deviation of 0.3%, while the CBCT‐based calculation showed a mean deviation of −4.6%. The gamma passing rates for the synthetic‐based calculation were 98.1%, 96.2%, and 96.1% for axial, sagittal, and coronal slices, respectively, while the gamma passing rates for the CBCT‐based calculation were 96.2%, 89.4%, and 89.4%, respectively.

Table 7 shows 2D axial gamma passing rates for the test patients. On average, the mean dose difference between the synthetic‐based calculation and the CTdef‐based calculation was 0.44% while the CBCT‐based calculation showed a mean deviation of 1.06%. The average gamma passing rate with a 3%/2 mm criteria for the synthetic‐based calculation was 98.35% while the average passing rate for the CBCT‐based calculation was 96%.

**TABLE 7:** Two‐dimensional (2D) axial gamma passing rates (%) for the test group at isocenter

	P‐1	P‐2	P‐3	P‐4	P‐5	P‐6
CBCT‐CTdef	96.2	98.1	99.2	94.4	93.9	96.3
Synthetic‐CTdef	98.1	98.4	100	97.2	98.4	98.0

Abbreviations: CBCT, cone beam computed tomography; CTdef, deformed computed tomography; P, patient.

#### 4.2.4 Three‐dimensional gamma analysis

**FIGURE 9:**
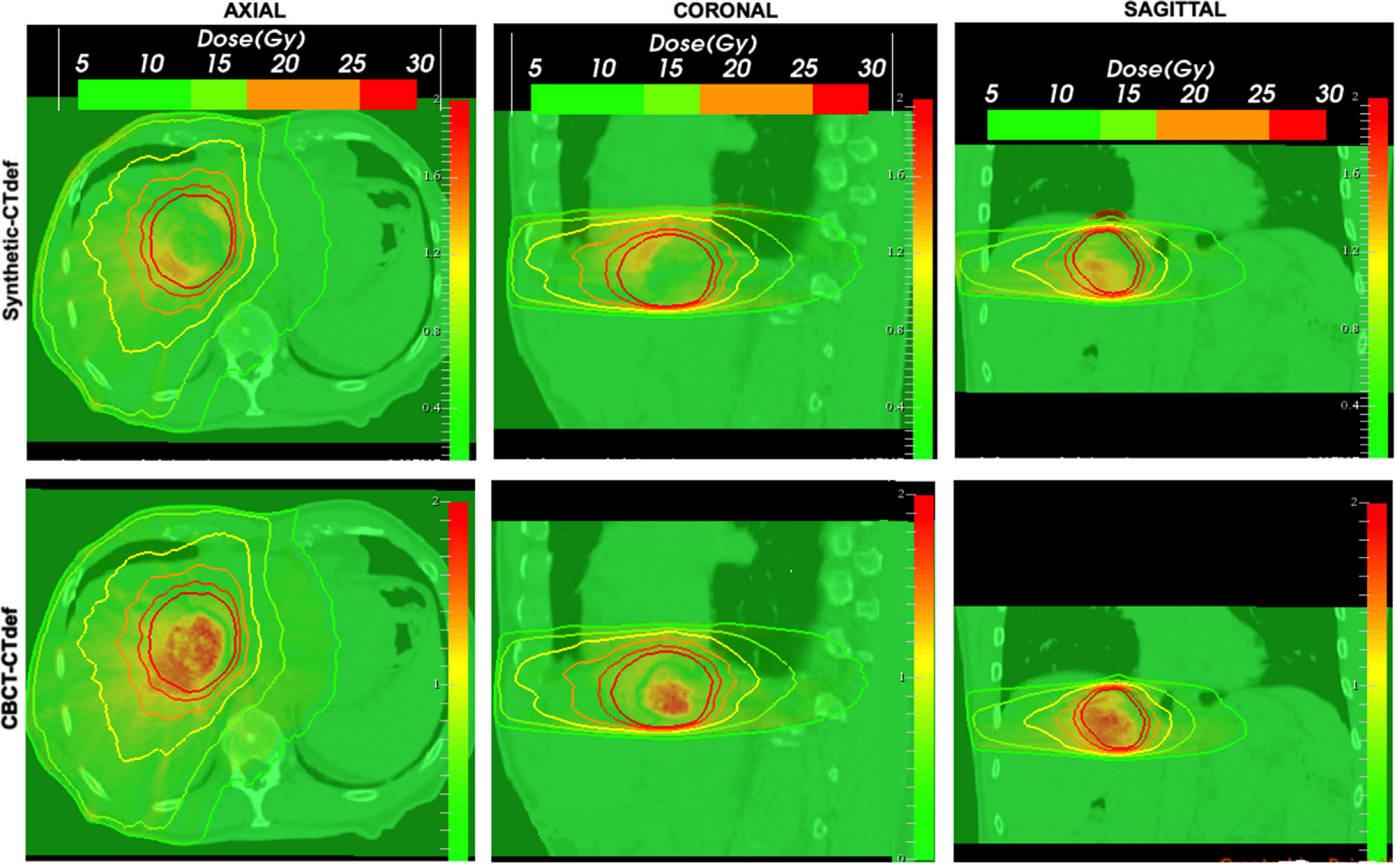
Synthetic (top) and cone beam computed tomography (CBCT) (bottom) three‐dimensional (3D) gamma maps overlaid on deformed computed tomography (CTdef) and reference isodose lines. Failing points for the 3%/2 mm criteria are shown for γ > 1.

The gamma analysis is most used for planar dose comparisons, however, here we used the 3D gamma implementation of the Low's method^14^ in the Slicer RT module^15^ to compare 3D dose volumes. CBCT, synthetic, and CTdef‐based dose maps were resampled, resized, and cropped to match the smallest dose matrix size (CBCT). Figure 9 shows gamma comparison plots for the synthetic‐based calculation and the CBCT‐based calculation using CTdef as the reference dose distribution for one of the test patients. A 3%/2 mm criteria with a 10% dose threshold was used for this comparison. Gamma plots for the rest of the patients can be found in the Supporting Information.

Table 8 shows 3D gamma passing rates for the test patients. The average gamma passing rate with a 3%/2 mm criteria for the synthetic‐based calculation was 98.95%, while the average passing rate for the CBCT‐based calculation was 98.97%.

**TABLE 8:** Global three‐dimensional (3D) gamma passing rates (%) for the test group

	P‐1	P‐2	P‐3	P‐4	P‐5	P‐6
CBCT‐CTdef	95.18	100	100	99.94	98.89	100
Synthetic‐CTdef	94.11	100	100	99.97	99.64	100

Abbreviations: CBCT, cone beam computed tomography; CTdef, deformed computed tomography; P, patient.

The 3D gamma maps were overlaid on the reference image showing the reference isodose lines. Gamma values were given an upper bound of 2. The 3D gamma maps show that the synthetic‐based calculation has overall better agreement with the reference calculation. However, for one of the test patients (P1), there is one area of concern near the right lung‐liver interface which corresponds to the 5 Gy isodose mismatch observed before. The gamma passing rate for this patient's synthetic‐based dose distribution was 94.11%.

**FIGURE 10:**
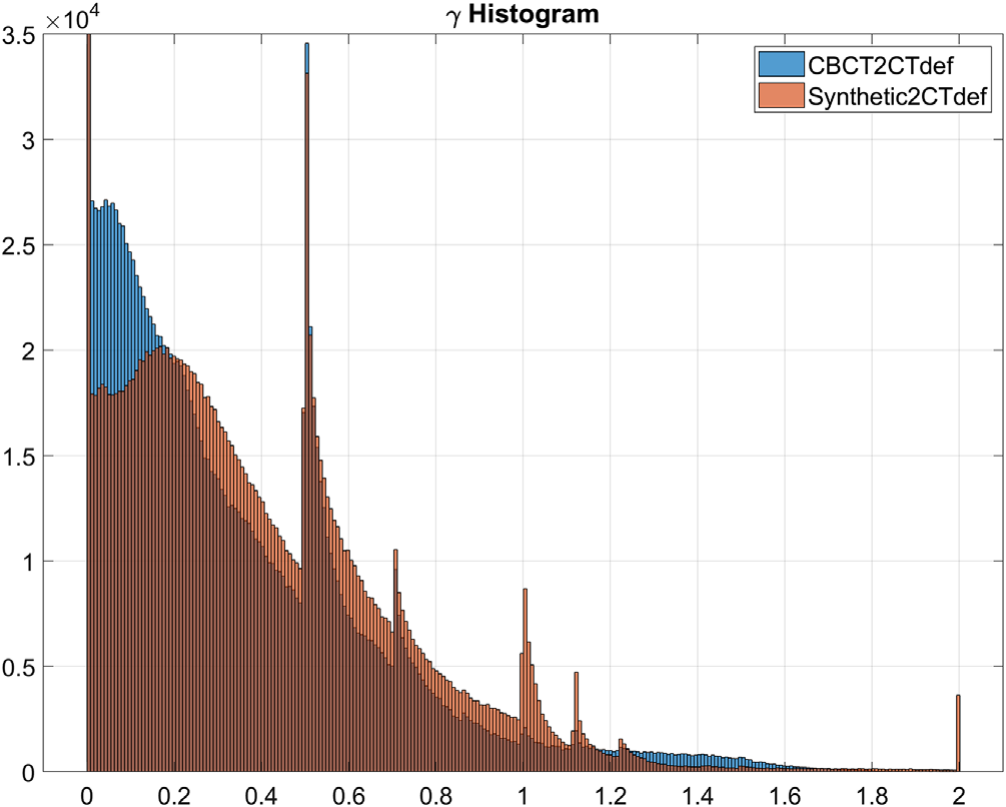
Synthetic (orange) and cone beam computed tomography (CBCT) (blue) gamma histograms. 256 bins. The vertical axis represents the count (frequency) and the horizontal axis represents the gamma range.

**FIGURE 11:**
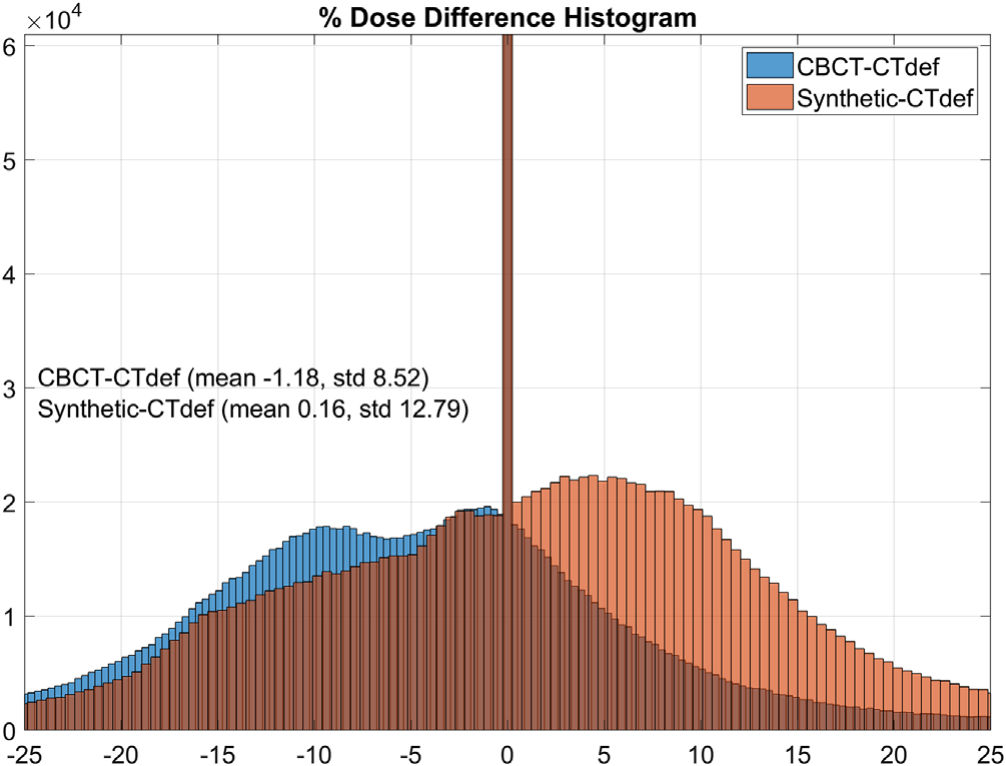
Synthetic (orange) and cone beam computed tomography (CBCT) (blue) percent dose difference histograms, 10% threshold. 1024 bins. The vertical axis represents the count (frequency) and the horizontal axis represents the percent dose difference range.

Interestingly, despite showing widespread disagreement, the CBCT‐based distribution had a passing rate of 95.18%. To understand this incongruity, we plotted the gamma histograms for the synthetic‐based and the CBCT‐based dose distributions on Figure 10 and the percent dose difference histograms in Figure 11.

In the passing range (0 < γ < 1), the CBCT‐CTdef gamma volume has 1.07% more voxels than the synthetic‐CTdef gamma volume; however, the synthetic‐CTdef gamma volume had more voxels for 0 < γ < 0.007 and at every bin range except for 0.015 < γ < 0.2. The synthetic‐CTdef gamma volume also showed a spike in voxels with 1.993 < γ < 2 which contributed to the lower passing rate.

On the other hand, the percent dose difference histogram shown in Figure 11 illustrates that the mean percent dose difference between the CBCT‐based dose calculation and CTdef is −1.18%, while the mean percent dose difference between the synthetic‐based dose and CTdef was 0.16%. These results correlate with what was presented before on the DVH and dose profile comparisons where underestimation of the dose was evident for the CBCT‐based calculation.

## 5 DISCUSSION

A successful implementation of adapting planning relies on the accuracy of the feedback imaging acquired during daily treatment. Because of the inherent limitations of CBCT images, which are the result of a compromise between image quality, acquisition speed, and dose to the patient, adaptive treatments have depended on image processing to compensate for such limitations. Up to the present time, the favored adaptive method in the clinical practice is to deform the planning CT image and its contours to match the daily acquired CBCT, then proceed to re‐calculate the dose for adaptive monitoring or re‐planning.^1^

Despite the undeniable benefits of the multiple uses of deformable image registration in radiotherapy planning, some limitations persist making the process overly user‐dependent. During a treatment course, the body's anatomy might go through changes that cannot be considered as small deformations of the original volume but the addition or absence of entirely new structures such as air pockets and mass growths. To force the deformable registration algorithm around these challenges, tools such as structure‐guided deformation^16^ can be used; however, achieving a good fit for certain structures is accompanied by a detrimental fit for others. Even commercially available software for adaptive planning requires the user to manually correct the deformed contours adding a significant amount of time to the adaptive planning process.

With this study, we assessed an alternative to deformable image registration based on synthetically generated images using deep learning algorithms. We postulated that synthetically generated images can produce comparable results to a deformed CT in terms of dose calculation while displaying a more accurate representation of the daily anatomy and therefore improving the dosimetric accuracy. Additionally, the generation of synthetic images is not user‐dependent and can be completed relatively faster than deformable image registration once the training stage is finalized.

To support our proposition, first we assessed the accuracy of the synthetic images regarding the preservation of anatomical structures, image quality, and HU accuracy. The synthetically generated images exhibited a noteworthy increase in image quality compared to the CBCT image. Increased image quality was demonstrated through increased SNR, contrast resolution and lower RMSE, MAE, noise, and artifact severity. Superior edge matching, sharpness, and preservation of anatomical structures from the CBCT image was observed for the synthetic images when compared to the deformable image registration method. Quantitative metrics, such as the Hausdorff distance and the Dice coefficient also showed a higher correlation between the synthetic image contours and CBCT contours when compared with those obtained through deformable registration. Furthermore, comparison of 3D volume HU histograms showed that synthetic images and CT images have similar HU histograms that significantly differ from the CBCT HU histogram. Organ‐based HU statistics showed that the mean percent HU difference from the CT images was 17.2% for the synthetic images and 42.8% for the CBCT images. Additionally, overall variability of the synthetic image correlates with the CT image variability while CBCT images showed a significantly increased variability.

We assessed the dosimetric accuracy of the synthetic‐based dose calculation through the analysis of DVHs, isodose lines comparison, and gamma index evaluation. Three verification plans (CBCT, CTdef, and synthetic) were created from the original treatment plan and DVHs statistics were calculated. The largest difference across the three plans was observed in the PTV. The average volume receiving the prescription dose was 92% for CTdef, 93% for synthetic, and 70% for CBCT. The synthetic‐based calculation showed comparable results to the CTdef‐based calculation with a maximum mean deviation of 1.5% for the DVH statistics and 0.44% for dose profiles, while the CBCT‐based calculation showed a maximum mean deviation of 3.6% for the DVH statistics and 1.06% for the dose profile statistics. To add spatial information to the dosimetric accuracy assessment, isodose volumes were calculated and represented on the orthogonal planes. Overall, synthetic‐based isodose lines showed better agreement to the CTdef‐based isodose lines than the CBCT‐based. An interesting case was presented highlighting some limitations near the air‐tissue interface. Motion artifacts were significantly higher in this region, with the averaging effect being more pronounced on the CBCT image. The deep learning algorithm produced images with sharper edges, therefore HU values in air near air‐tissue interfaces were consistently lower than the ones expected in a real CT image.

While the current clinical practice and the literature indicate that the passing rate of the gamma criteria is commonly used to assess the level of agreement between two dose distributions,^17^ it should be noted that the efficacy of this method had to be placed under scrutiny in our study when the CBCT‐based dose distribution passed the 3D gamma test despite evident dose underestimation shown in the DVH analysis. Gamma maps showed that the synthetic‐based calculation had overall better agreement with the CT‐based calculation; however, the gamma passing rate for the synthetic‐based dose distribution and the CBCT‐based dose distribution were 94.11% and 95.18%, respectively, for 3%/2 mm and 10% threshold criteria. Recent studies have questioned the efficacy of gamma passing rates in comparing two dose distributions.^18^, ^19^ Furthermore, two studies^20^, ^21^ found that the gamma criteria had a low sensitivity in detecting clinically relevant plan errors. Although the efficacy of the gamma test is not the subject of study of this paper, it is important for the medical physics community to keep questioning the gamma passing rate alone as an effective tool to compare dose distributions for patient quality assurance among other uses.

Methods to leverage synthetized medical images for adaptive planning have been described before in the literature. Some have focused on enhancement and artifact removal of the CBCT images^10^, ^13^ while others have focused on improving CBCT segmentation.^22^, ^23^ Our approach differs from prior studies in that we have focused on the accuracy of the synthetic images for dose calculation and how the currently used method based on deformable image registration can be improved. While most studies have focused on head and neck or prostate except for Liu et al.,^24^ that studied the pancreatic region, we implemented our methodology in an anatomical region with high inter and intra‐fraction variability and with a wide HU profile such as the upper abdominal area. These are the areas that challenge the image registration algorithms the most, therefore here we assessed whether synthetically generated images would be able to overcome these challenges.

Three technical limitations were identified in our study that could be the focus of future studies. First, the model is trained on images from a specific OBI‐CT simulator pair, hence the trained model is not generalizable to all systems. Second, the CycleGAN implementation uses single slices (2D) from the input volumes (3D). A 3D implementation will improve the performance of the model particularly for any slice‐to‐slice inconsistency^25^, ^26^; however, GPU memory will be the greatest challenge to overcome here. Lastly, the reduced field of view of CBCT images will limit the implementation of synthetic only‐based adaptive planning to small targets as the PTV and close organs at‐risk must be fully contained in the image. Workarounds for this limitation could be to fill in the missing data from the planning CT volume as a valid approximation.

## 6 CONCLUSIONS

Our findings show that GANs can produce reliable synthetic images for the adaptive delivery framework. The synthetically generated images can preserve the anatomical features of the CBCT images while matching the HU of the planning CT image within an acceptable range. Thus, dose calculations can be performed on the synthetic images with minimal error. Finally, enhanced image quality of the synthetic images could potentially translate into better daily alignment, increasing overall treatment delivery accuracy.

## CONFLICT OF INTEREST

The authors whose names are listed certify that they have no affiliations with or involvement in any organization or entity with any financial interest (such as honoraria; educational grants; participation in speakers’ bureaus; membership, employment, consultancies, stock ownership, or other equity interest; and expert testimony or patent‐licensing arrangements) or nonfinancial interest (such as personal or professional relationships, affiliations, knowledge, or beliefs) in the subject matter or materials discussed in this manuscript.

## AUTHOR CONTRIBUTIONS

Olga M. Dona Lemus designed the project, acquired and analyzed the data, and wrote the manuscript. Yi‐Fang Wang and Li Feng contributed to data acquisition and analysis and revised the manuscript. David P. Horowitz, Yuanguang Xu, and Sachin Jambawalikar made significant contribution to the design of the work and revised the manuscript. Cheng‐Shie Wuu provided the resources needed for the project, contributed to the conception and design of the work, and revised the manuscript. All authors approved the submitted draft and agreed to be accountable for all aspects of this work.

## Supporting information

Supporting information

Supporting information
