# Supplementary material for: Dosimetric assessment of patient dose calculation on a deep learning‐based synthesized computed tomography image for adaptive radiotherapy
Source: J Appl Clin Med Phys. 2022 Mar 25;23(7):e13595. doi: 10.1002/acm2.13595 (PMC9278692; doi:10.1002/acm2.13595)
Supplement: Supplementary file 1 — Supporting information [file ACM2-23-e13595-s002.docx]

**Supplementary Material**

**Loss functions**

The following are the loss functions implemented in our study. Cycle consistency and adversarial loss were implemented without modification as optimized by the original method from Zhu et al [9]. To better achieve our goal for preservation of anatomical features from the CBCT image and HU numbers from the CT image, we added the four additional loss functions proposed by Kida et al. [13]. The gradient loss (loss_grad_) encourages structural preservation by conserving the image edges. Idempotent loss (loss_idem_) ensures that the generators are unchanged in value when multiplied or operated on by itself. Finally, the total variation regularization loss (loss_tv_) forces the generator to produce spatially uniform images.

${loss}_{Discriminator}=\lambda_{D}\sum_{x\in CBCT} \left( {\|D_{CT}\left( G_{CBCT\to CT}\left( x \right) \right)-0\|}_{2}+{\|D_{CBCT}\left( x \right)-1\|}_{2} \right)+\lambda_{D}\sum_{y\in CT} \left( {\|D_{CBCT}\left( G_{CT\to CBCT}\left( y \right) \right)-0\|}_{2}+{\|D_{CT}\left( x \right)-1\|}_{2} \right)$

${loss}_{generator}=\lambda_{cycle}{loss}_{cycle}+\lambda_{adv}{loss}_{adv}+\lambda_{grad}{loss}_{grad}+\lambda_{idem}{loss}_{idem}+\lambda_{air}{loss}_{air}+\lambda_{tv}{loss}_{tv}$

${loss}_{cycle}=\sum_{x\in CBCT} \left( {\|{x-G}_{CT\to CBCT}\left( G_{CBCT\to CT}\left( x \right) \right)\|}_{1} \right)+\sum_{y\in CT} \left( {\|{y-G}_{BCT\to CT}\left( G_{CT\to CBCT}\left( y \right) \right)\|}_{1} \right)$

${loss}_{adv}=\sum_{x\in CBCT} \left( {\|D_{CT}\left( G_{CBCT\to CT}\left( x \right) \right)-1\|}_{2} \right)+\sum_{y\in CT} \left( {\|D_{CBCT}\left( G_{CT\to CBCT}\left( y \right) \right)-1\|}_{2} \right)$

${loss}_{grad}=\sum_{x\in CBCT}$

${loss}_{idem}=\sum_{y\in CBCT} \left( {\|{G_{CT\to CBCT}-G}_{CT\to CBCT}\left( G_{CT\to CBCT}\left( y \right) \right)\|}_{1} \right)+\sum_{x\in CT} \left( {\|{G_{CBCT\to CT}-G}_{CBCT\to CT}\left( G_{CBCT\to CT}\left( x \right) \right)\|}_{1} \right)$

${loss}_{tv}=\sum_{x\in CBCT} \left( {\|\nabla\left( G_{CBCT\to CT}\left( x \right) \right)\|}_{1} \right)$

**Deformable Image Registration verification**

The deformable image registration (CT-to-CBCT) used as the reference image in this study was done in Velocity (Varian Medical Systems, Inc, Palo Alto, CA). We used the daily shift as the rigid registration step and then proceeded with multistep deformable registration. The target registration error (TRE) was used to assess the deformable image registration process. Ten points were selected for TRE measurements including a mix of soft tissue and bony landmarks (Figure 12). All points were selected on the rigid registration and TRE was assessed on the deformable registration. Tables 8 and9 show the point detailed statistics and the TRE summary, respectively. The overall mean TRE was 3.0 ± 2.1 mm. The Jacobian map of the deformation was superimposed with the deformed image on Figure 13 to indicate topology changes in the image. Figure 14 shows the highest gradients around the air pockets, still the algorithm was unable to fully eliminate the air pocket from the image.

| 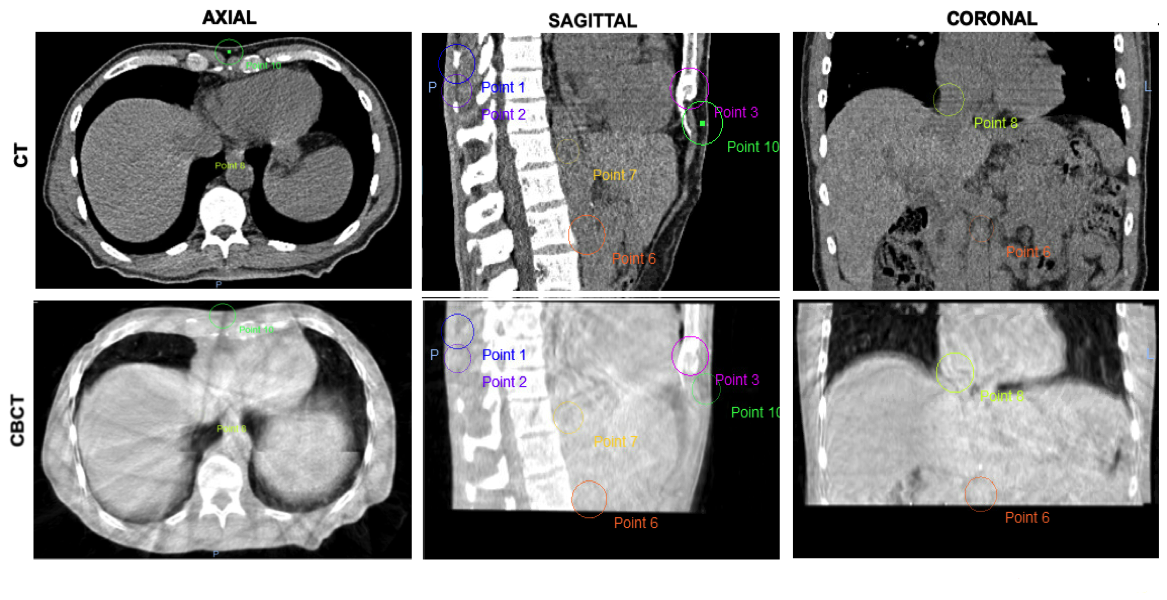 |
| --- |
| **Figure 12**. Location of the points used for the TRE calculation. The points were independently selected on the CT (top) and CBCT (bottom) images. |

| 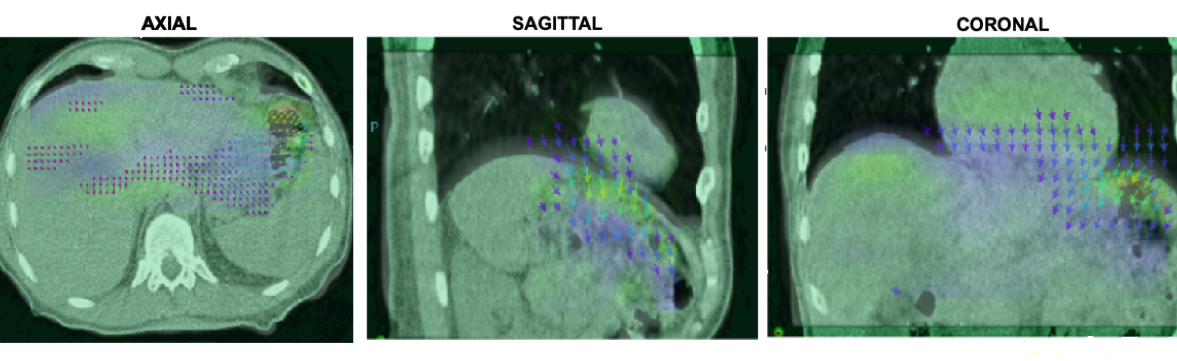 |
| --- |
| **Figure 13**. Determinant of the Jacobian deformation. Jacobian overlaid on the Axial, Coronal and Sagittal slices for the registered CBCT and CT images. Arrows pointing towards the direction of the deformation. |

| **Table 8.** Statistics Summary(mm) | | | | | | |
| --- | --- | --- | --- | --- | --- | --- |
|  | **Sum** | **Mean** | **Std dev** | **Median** | **Min** | **Max** |
| √(Δx²+Δy²+Δz²) | 29.6 | 3.0 | 2.1 | 2.4 | 0.5 | **7** |
| Δx | -1.6 | -0.2 | 2.3 | 0.1 | -4.1 | 2.9 |
| Δy | 0.4 | 0.0 | 2.2 | 0.2 | -3.8 | **4.9** |
| Δz | 3.6 | 0.4 | 1.7 | 0.4 | -4.2 | 2 |

| **Table 9.** Point Detailed Statistics (mm) | | | | |
| --- | --- | --- | --- | --- |
| **Point ID** | **Δx** | **Δy** | **Δz** | **√(Δx²+Δy²+Δz²)** |
| 1 | -1.0 | 1.0 | 0.0 | 1.4 |
| 2 | -1.0 | 1.0 | 0.0 | 1.4 |
| 3 | 0.0 | 0.5 | 0.0 | 0.5 |
| 4 | 1.0 | 0 | 2.0 | 2.2 |
| 5 | -3.9 | 4.9 | 2.0 | **6.6** |
| 6 | 1.9 | -1.0 | 2.0 | 3.0 |
| 7 | 0.3 | 1.0 | 0.8 | 1.3 |
| 8 | -4.1 | -3.8 | -4.2 | **7.0** |
| 9 | 2.3 | -1.2 | -0.1 | 2.6 |
| 10 | 2.9 | -1.9 | 1.0 | 3.7 |

| 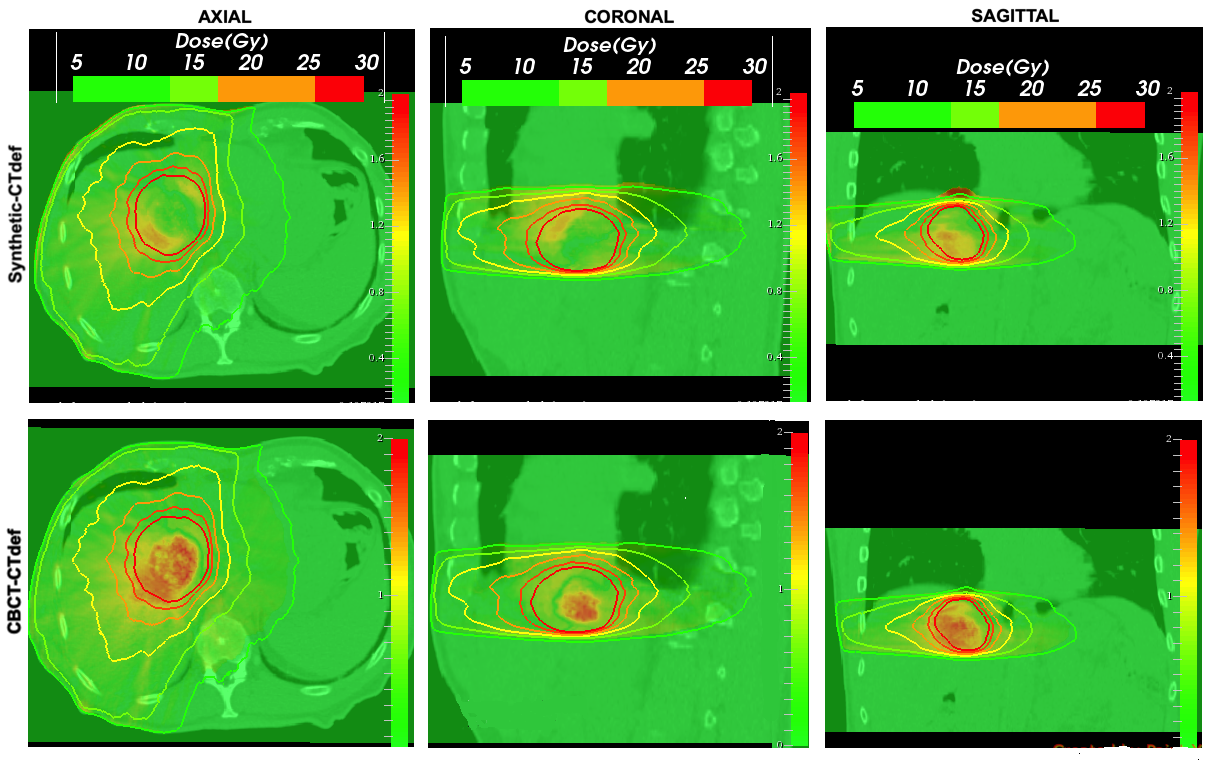 |
| --- |
| **Figure 14. P1 -** Synthetic (top) and CBCT (bottom) 3D gamma maps overlaid on CTdef and reference isodose lines. Failing points for the 3%/2mm criteria are shown for γ >1 |

| 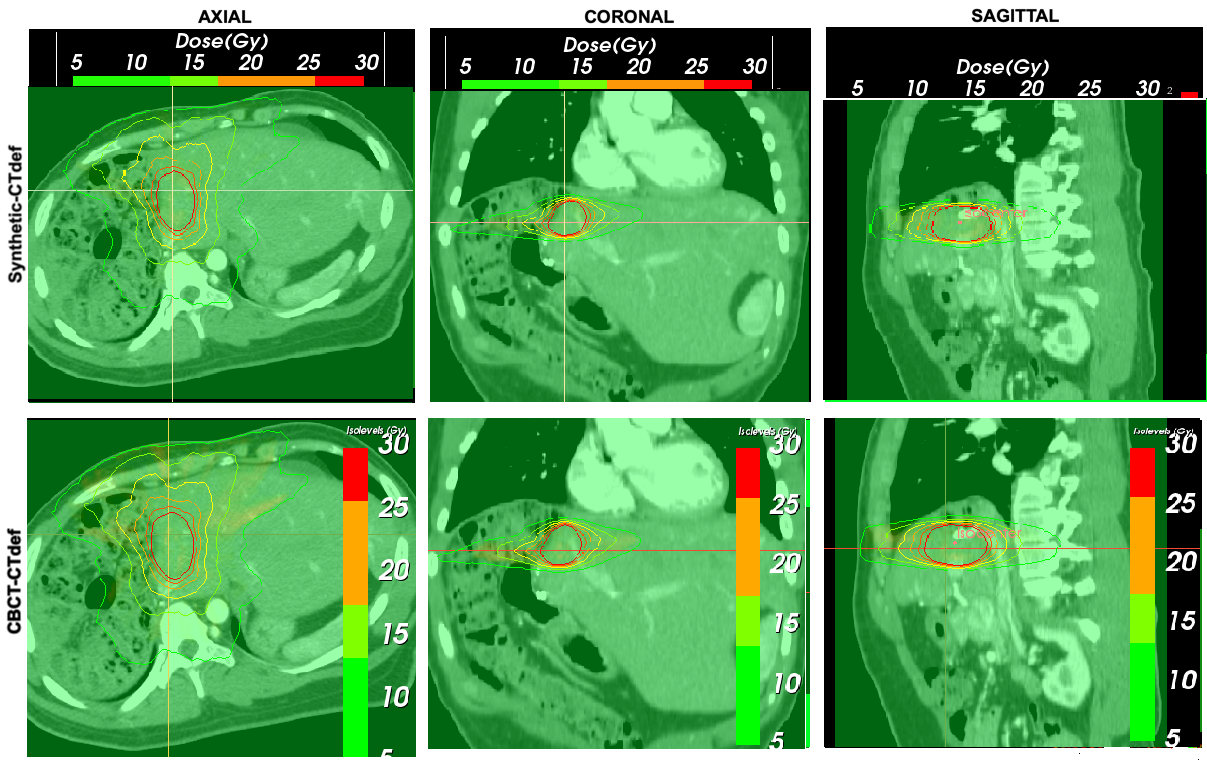 |
| --- |
| **Figure 15. P2 -** Synthetic (top) and CBCT (bottom) 3D gamma maps overlaid on CTdef and reference isodose lines. Failing points for the 3%/2mm criteria are shown for γ >1 |

| 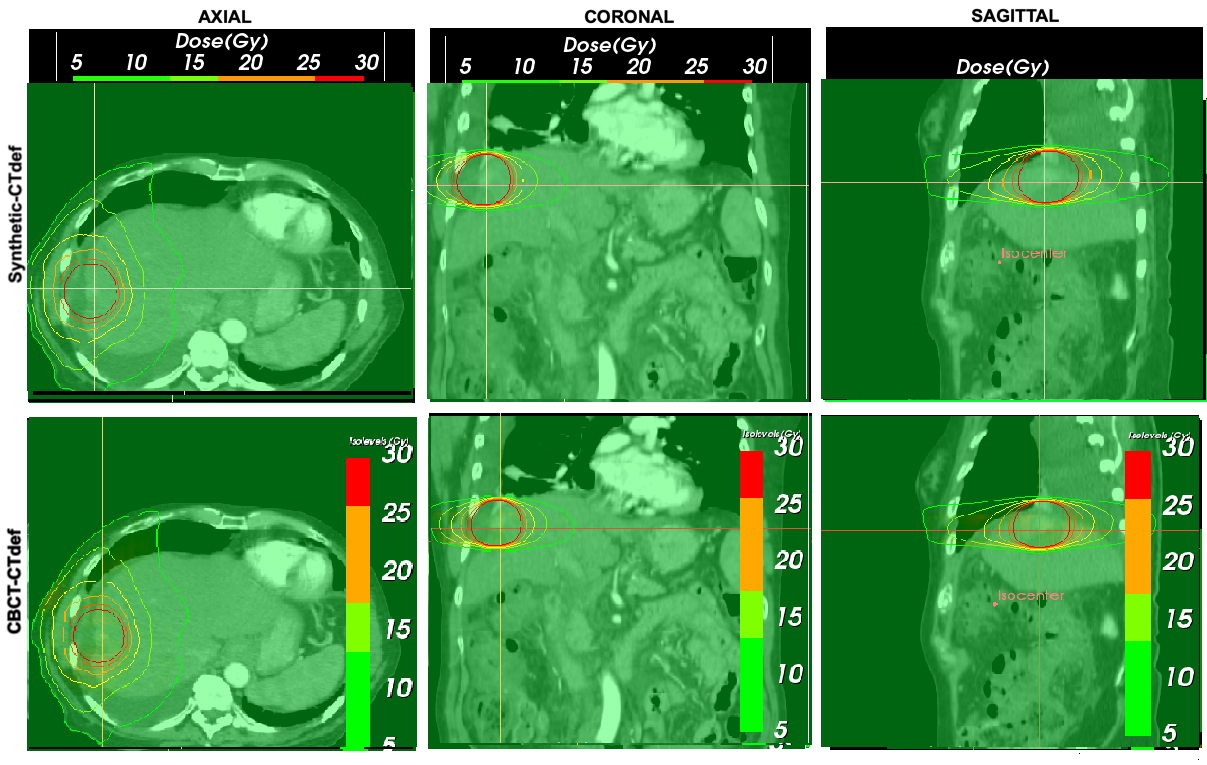 |
| --- |
| **Figure 16. P3 -** Synthetic (top) and CBCT (bottom) 3D gamma maps overlaid on CTdef and reference isodose lines. Failing points for the 3%/2mm criteria are shown for γ >1 |

| 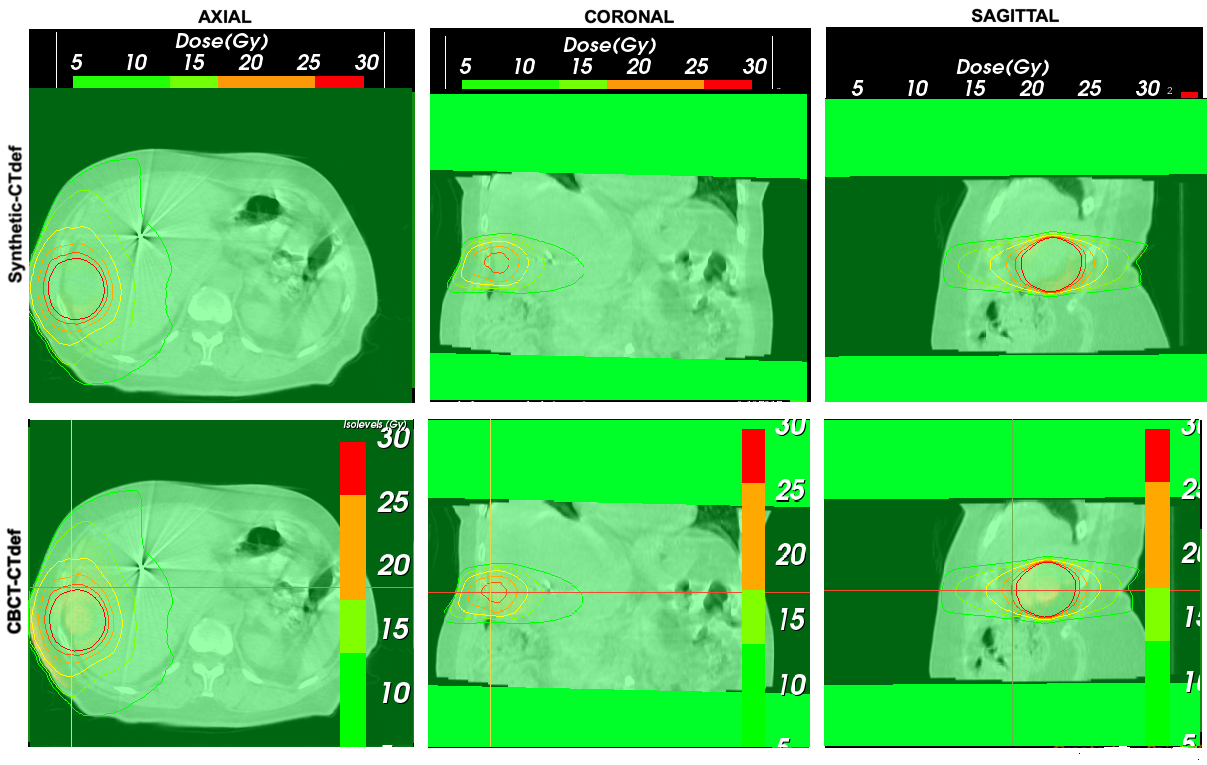 |
| --- |
| **Figure 17. P4 -** Synthetic (top) and CBCT (bottom) 3D gamma maps overlaid on CTdef and reference isodose lines. Failing points for the 3%/2mm criteria are shown for γ >1 |

| 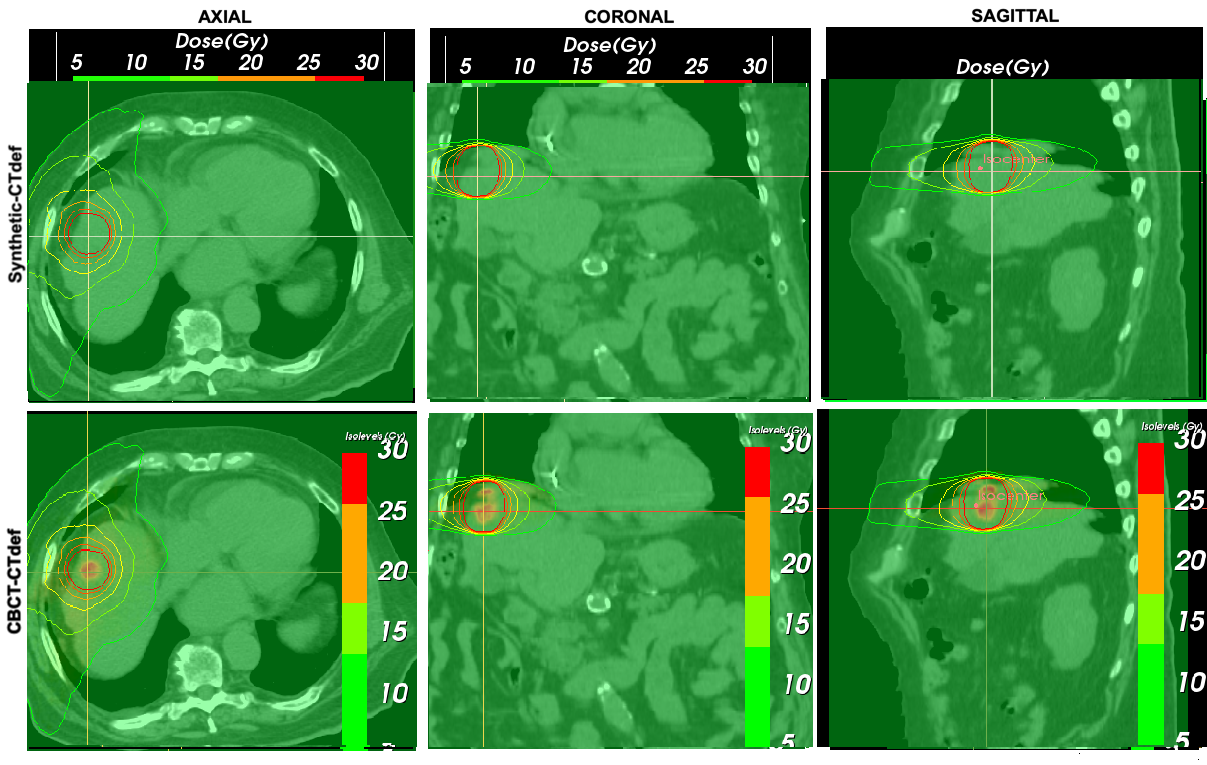 |
| --- |
| **Figure 18. P5 -** Synthetic (top) and CBCT (bottom) 3D gamma maps overlaid on CTdef and reference isodose lines. Failing points for the 3%/2mm criteria are shown for γ >1 |

| 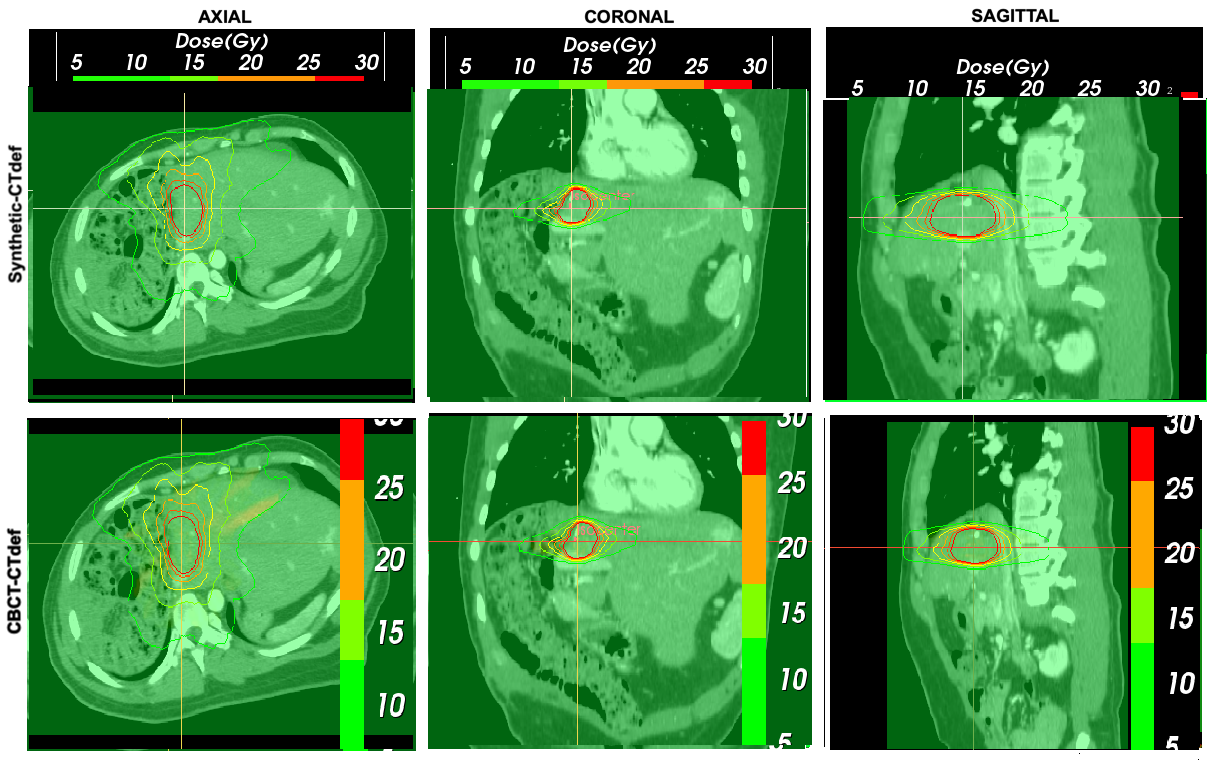 |
| --- |
| **Figure 19. P6 -** Synthetic (top) and CBCT (bottom) 3D gamma maps overlaid on CTdef and reference isodose lines. Failing points for the 3%/2mm criteria are shown for γ >1 |

| 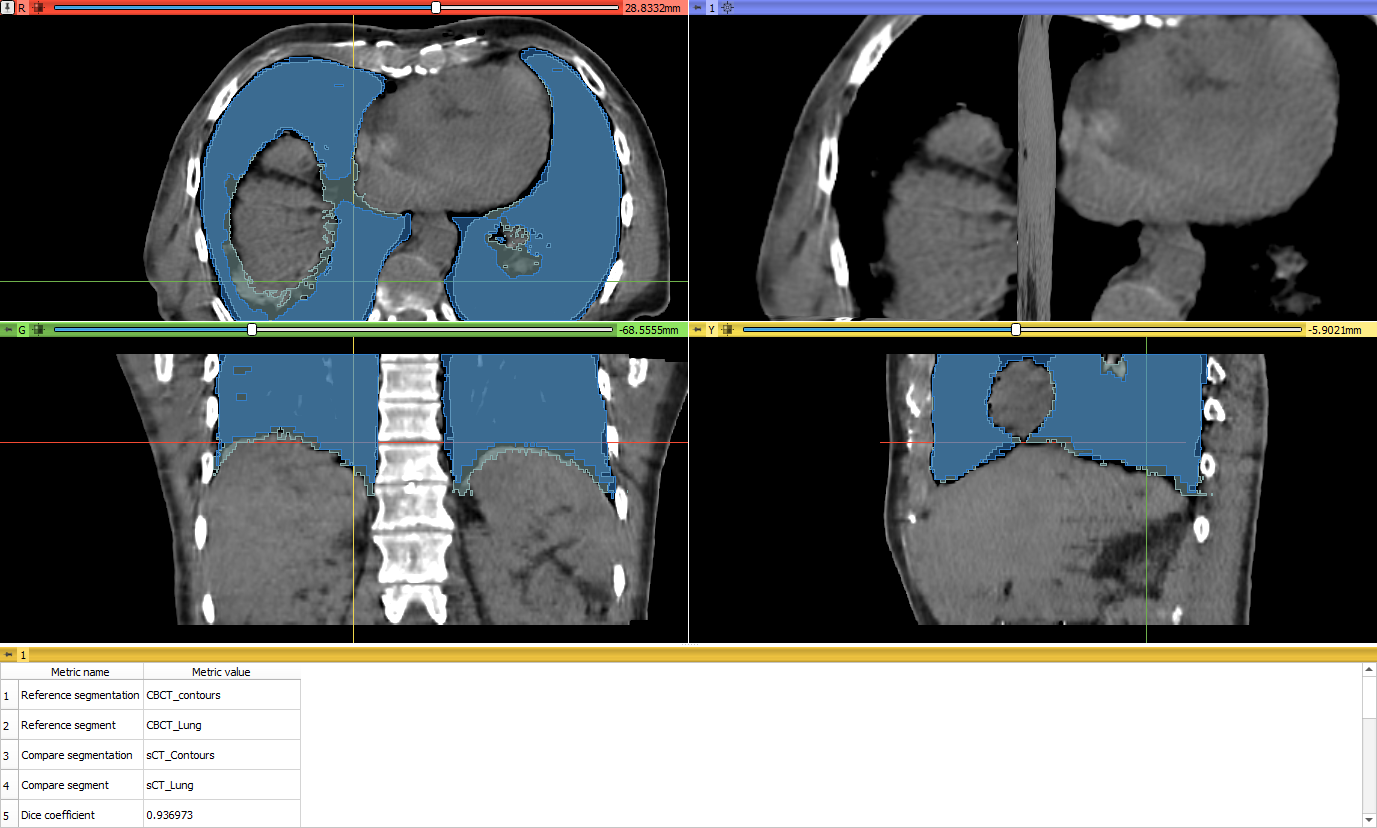 |
| --- |
| **Figure 20.** CBCT lung contour (light blue) and Synthetic CT (dark blue) lung contour overlay and associated dice coefficient for **P1**. Changes in HU at the interface were the main contributor to the reduced dice coefficient in lungs. Lung contours is generally created using image thresholding. |
