# Supplementary material for: Dosimetric assessment of patient dose calculation on a deep learning‐based synthesized computed tomography image for adaptive radiotherapy
Source: J Appl Clin Med Phys. 2022 Mar 25;23(7):e13595. doi: 10.1002/acm2.13595 (PMC9278692; doi:10.1002/acm2.13595)
Supplement: Supplementary file 2 — Supporting information [file ACM2-23-e13595-s001.docx]

**Title:** Dosimetric Assessment of Patient Dose Calculation on a Deep Learning Based Synthesized CT image for Adaptive Radiotherapy

**Running title:** Synthetic CT-Based Dose Calculation

**Contributing Authors:**

Olga M. Dona Lemus^1^, Yi-Fang Wang^1^, Li, Feng^1^, Sachin Jambawalikar^3^, David P Horowitz^1,2^ , Yuanguang Xu^1^, Cheng-Shie Wuu^1^

^1^Department of Radiation Oncology, Columbia University Irving Medical Center, New York, NY,

^2^Herbert Irving Comprehensive Cancer Center, New York, NY

^3^Department of Radiology, Columbia University Irving Medical Center, New York, NY

**Corresponding Author:** Olga M. Dona Lemus, Ph.D.

Department of Radiation Oncology

Columbia University Medical Center

CHONY North Bsmt Room 11

622 West 168th Street

New York, NY 10032

Tel: 646-317-0179

Email:omdona@gmail.com and od233@cumc.columbia.edu

**Senior Author:** Cheng-Shie Wuu, Ph.D.

**Keywords:** Synthesized CT, Deep Learning, Dosimetric accuracy

**Conflicts of Interest Statement:** The authors whose names are listed immediately below certify that they have NO affiliations with or involvement in any organization or entity with any financial interest (such as honoraria; educational grants; participation in speakers’ bureaus; membership, employment, consultancies, stock ownership, or other equity interest; and expert testimony or patent-licensing arrangements), or non-financial interest (such as personal or professional relationships, affiliations, knowledge or beliefs) in the subject matter or materials discussed in this manuscript.

**Author Contribution Statement:** OMDL designed the project, acquired, and analyzed the data and wrote the manuscript. YFW and FL contributed to data acquisition and analysis and revised the manuscript. DPH, YX and SJ made significant contribution to the design of the work and revised the manuscript. CSW provided the resources needed for the project, contributed to the conception and design of the work, and revised the manuscript. All authors approved the submitted draft and agreed to be accountable for all aspects of this work.
